# Mealworm larvae (*Tenebrio molitor*) and crickets (*Acheta domesticus*) show high total protein *in vitro* digestibility and can provide good-to-excellent protein quality as determined by *in vitro* DIAAS

**DOI:** 10.3389/fnut.2023.1150581

**Published:** 2023-07-03

**Authors:** Laila Hammer, Diego Moretti, Lychou Abbühl-Eng, Pabiraa Kandiah, Nikolin Hilaj, Reto Portmann, Lotti Egger

**Affiliations:** ^1^Agroscope Liebefeld-Posieux, Berne, Switzerland; ^2^Department of Health, Swiss Distant University of Applied Sciences (FFHS), Brig, Switzerland; ^3^Division of Human Nutrition, Wageningen University & Research, Wageningen, Netherlands; ^4^Department of Health Sciences and Technology, Human Nutrition Laboratory, ETH Zurich, Zurich, Switzerland

**Keywords:** digestibility, insects, *Tenebrio molitor*, *Acheta domesticus*, chicken, *in vitro* DIAAS, protein quality, food processing

## Abstract

Edible insects, such as mealworms (*Tenebrio molitor larvae*; TM) and crickets (*Acheta domesticus*; AD), are a sustainable, protein-dense novel food with a favorable amino acid profile, which might be an alternative to animal proteins. To assess the protein quality of TM and AD, we assessed the digestible indispensable amino acid scores (DIAAS), considering individual amino acids and their ileal amino acid digestibility, using an *in vitro* model based on the INFOGEST digestion protocol. In addition, we evaluated if various processing and food preparation steps influenced the *in vitro* digestibility of individual amino acids and the *in vitro* DIAAS values of TM and AD and compared them to chicken breast as a reference of excellent protein quality. The total protein *in vitro* digestibility ranged from 91 to 99% for TM and from 79 to 93% for AD and was negatively affected by oven-drying and, to a lesser extent, by chitin-reduction. The *in vitro* DIAAS values were 113, 89, and 92 for chicken, blanched TM, and blanched AD, respectively, when considering the indispensable amino acid (IAA) requirements of young children between 6 months and 3 years. Across different processing and food preparation methods, the *in vitro* DIAAS values ranged between 59 and 89 for TM and between 40 and 92 for AD, with the lowest values found in chitin-reduced insects. Due to their similarities to chicken regarding protein composition, total protein *in vitro* digestibility, and *in vitro* DIAAS values, TM and AD might be an alternative to traditional animal proteins, provided that suitable processing and food preparation methods are applied. Our *in vitro* DIAAS results suggest that TM and AD can thus be considered good-quality protein sources for children older than 6 months. The DIAAS calculations are currently based on crude protein (total nitrogen × 6.25), resulting in an overestimation of insect protein content, and leading to an underestimation and potential misclassification of protein quality. The *in vitro* model applied in this study is a valuable tool for product development to optimize the protein quality of edible insects. Further studies are required to assess the *in vivo* DIAAS of insects in humans.

## 1. Introduction

There is an urgent need for more sustainable protein sources to supply the growing global population within the current planetary constraints. The contribution of dietary habits to climate change is substantial, and transitioning towards more sustainable diets will require, at least in high-income countries, reduced meat consumption (1). As meat is a key source of high-quality protein, however, an uninformed dietary shift could put vulnerable population groups at an increased risk of nutritional deficiencies. Some edible insects have been proposed as suitable alternatives to meat because of their amino acid (AA) profiles, their high protein content, and their potential for mass production (2) at a reduced burden on the environment. Compared to livestock, insect farming has a lower environmental impact (3, 4) because of more favorable feed conversion ratios (5, 6), greenhouse gas emissions (7), water pollution (8), and land use (9). To date, more than 2,000 edible insects have been reported (10), which are consumed by at least 2 billion people (11) as part of their traditional diets (12). In recent years, novel food regulations for mealworms (*Tenebrio molitor larvae*; TM), crickets (*Acheta domesticus*; AD), grass hopers (*Locusta migratoria*), and lesser mealworm (*Alphitobius diaperinus*) were introduced in Switzerland and the EU, which defined these species as novel foods for human consumption (13–16). However, the protein quality of these species and their adequacy to meet the dietary indispensable amino acid (IAA) requirements of humans have not been assessed systematically.

Food protein quality is defined by AA composition and digestibility of AA, relative to human IAA requirements (15). The digestible indispensable amino acid score (DIAAS) is recommended by the Food and Agriculture Organization of the United Nations (FAO) (17) to evaluate protein quality and replaces the protein digestibility-corrected amino acid score (PDCAAS). DIAAS assessment should be based on the digestibility and bioavailability of individual AA at the end of the small intestine, and as the data on ileal IAA digestibility of foods determined in humans are limited (17), *in vitro* models to mimic digestion (18, 19) and predict protein digestibility and IAA bioavailability in humans are needed (17). Recently, we reported an *in vitro* model to determine the predicted true ileal protein digestibility at the level of individual AA and estimated DIAAS (20) by applying the static INFOGEST *in vitro* digestion (IVD) (18, 19) validated with *in vivo* data from humans and pigs for seven substrates (20). With this model, it is possible to screen novel foods for their protein quality.

The nutrient composition of edible insects can vary considerably, as it is influenced by their development stage, the region, the rearing substrate composition, the harvesting conditions (season, wild or commercially reared) (9, 21, 22), and food processing (12, 23, 24). Nonetheless, the IAA profiles of TM and AD are promising for human nutrition (14, 25) and meet or exceed dietary IAA requirements (26); however, the ileal IAA digestibility of TM and AD have not yet been assessed in humans. In growing pigs, Malla et al. (27) reported standardized ileal IAA digestibility ranging from 63.2 to 96.3% for TM and from 78.7 to 96.7% for AD in diets consisting of 10% insect crude protein. For another insect species belonging to the same order as TM (Coleoptera), Hermans et al. (28) found no difference in muscle protein synthesis after the intake of lesser mealworm larvae (*Alphitobius diaperinus*) and milk-derived protein in humans.

The protein digestibility of edible insects is expected to vary considering the high variability in reported IAA composition (12). The impact of processing, drying, and food preparation methods on protein digestibility may also affect the protein quality of TM and AD. In Europe, edible insects that are permitted as novel foods will not be consumed in their raw state, as legislation requires at least some type of heat treatment (13, 29, 30) to reduce the microbiological risks (31). The influence of food processing and preparation methods on protein digestibility cannot be easily predicted in most cases, as multiple factors can simultaneously be affected. For example, the concentration of anti-nutritional factors, such as polyphenols (32) and chitin (33), could interact with the proteins themselves or with the digestive enzymes, thus inhibiting their activity. Additionally, heat treatment can alter the structure of proteins, which often improves, but sometimes reduces, the digestibility of individual IAA (34), as protein-crosslinks (35) and covalent aggregates caused by the Maillard browning reaction (36) occur. Moreover, AA may be modified and their bioaccessibility changed, depending on the conditions during processing, resulting in the racemization of AA to D-enantiomers (37), the destruction of heat-sensitive AA, or the oxidation of some AA (38). Consequently, identifying the optimal processing methods for specific insect species to preserve food quality and maintain palatability are important steps towards consumer acceptability of edible insects (11).

To better understand the link between food processing and protein quality, this study aimed 1) to identify and characterize the proteins in TM and AD undergoing processing and food preparation methods and to describe the kinetics of protein hydrolysis by measuring the peptides liberated during IVD from selected proteins of TM, AD, and chicken, 2) to determine the total protein *in vitro* digestibility, *in vitro* digestibility of individual AA, and *in vitro* DIAAS in TM and AD undergoing different processing and food preparation methods, and 3) to evaluate their protein quality in comparison to cooked chicken breast, used as a reference.

## 2. Materials and methods

### 2.1. Chemicals, reagents, and substrates

For this study, all the reagents and enzymes were purchased from Merck (Zug, Switzerland). The analyzed substrates are listed in Table 1. Insects used in this study were obtained from Insekterei GmbH (registered in Switzerland as UID CHE-141.641.289) specialized in producing edible insects. The Swiss legislation does not require review and approval by an ethics committee because the insects were produced for human consumption according to Swiss food law, and available for sole in Swiss supermarkets. Insect rearing and preparation steps, were all performed at Insekterei and consisted of a 24-h fasting period before euthanasia, which was performed by freezing at −18°C for 24 h. The subsequent main processing steps are summarized in Table 1. The blanching of 1 kg of TM and AD was performed in water of minimally 88°C for 90 s and 93°C for 150 s, respectively. The commercially obtained freeze-dried TM and freeze-dried AD were processed according to the procedure of Hilaj et al. (39) to produce chitin-reduced powders. Briefly, freeze-dried substrates were ground in a food blender (Philips HR3655/00, Netherlands) with ultrapure water (1,2 w/w) and filtered through a cheesecloth. The resulting solution was freeze-dried (Freeze Drying Plant Sublimator 3–4-5/15, Zirbus Technology Benelux B.V., Netherlands) and ground into a fine powder (A 11 basic analytical mill, Germany).

**Table 1:** Overview of analyzed substrates and most critical processing steps.

Substrates	Processing procedure
*T. molitor* larvae, raw	Unprocessed and stored vacuum-packed at −18°C as whole insects^a^
*T. molitor* larvae, blanched	Blanched and stored vacuum-packed at −18°C as whole insects^a^
*T. molitor* larvae, freeze-dried	Blanched and freeze-dried as whole insects^b^
*T. molitor* larvae, pulverized	Blanched, freeze-dried,^b^ and ground into a fine powder^c^
*T. molitor*, chitin-reduced	Blanched, freeze-dried,^b^ chitin-reduced, and ground into a fine powder^c^
*A. domesticus*, raw	Unprocessed and stored vacuum-packed at −18°C as whole insects^a^
*A. domesticus*, blanched	Blanched and stored vacuum-packed at −18°C as whole insects^a^
*A. domesticus*, oven-dried	Blanched, oven-dried at 90°C to achieve drying loss of 65%, stored vacuum packed as whole insects^a^
*A. domesticus*, freeze-dried	Blanched and freeze-dried^b^
*A. domesticus*, pulverized	Blanched, freeze-dried,^b^ and ground into a fine powder^c^
*A. domesticus*, chitin-reduced	Blanched, freeze-dried,^b^ chitin-reduced, and ground into a fine powder^c^
Chicken breast, cooked	Cooked in skillet for 10 min and stored vacuumed at −18°C^d^

a Rearing and processing carried out at Insekterei GmbH, Switzerland.

b Freeze-dried insects obtained commercially (Migros, Switzerland).

c Processing performed at ETH, Switzerland.

d Unprocessed meat obtained commercially (Coop, CH) and prepared at Agroscope, Switzerland.

### 2.2. Composition of substrates

Dry matter was determined by the difference in the original weight and drying loss, measured using ISO standard 5,534:2004 (40). Fat content was analyzed gravimetrically according to Schmid-Bondzynski (41). Total nitrogen (TN) was determined by the Kjeldahl method (42). At the current status, no ISO methods exist including insects in the scope, therefore, ISO methods with alternative matrix scope, such as for dairy products (43–45), or for animal feed stuff (46) were applied. Total amino acids (TAA) were measured with the AOAC method 2018.06 for infant formula (47), with the modifications described in Sousa et al. (20), but the acid hydrolysis time of 24 h was followed for the substrates, as the hydrolysis time of 15 h was only applied to *in vitro* digestion samples. The tryptophan content was determined according to ISO 13904:2014 (48). The chitin content of freeze-dried and chitin-reduced substrates was determined as described in Hilaj et al. (39). All the parameters were determined in duplicates except for TAA analysis, which was conducted in triplicates.

By convention, protein content is calculated by multiplying TN by 6.25 as the standard, default nitrogen-to-protein conversion factor (NPCF) (49, 50). However, nitrogen derived from chitin in the form of N-acetylglucosamine contributes to TN and thus results in an overestimation of insect protein (51). Therefore, protein content was assessed as crude protein (TN × 6.25) and calculated as the sum of anhydrous AA.

### 2.3. Protein identification (extraction, SDS-PAGE, LC–MS)

To identify insect proteins, proteins were extracted from the substrates and subsequently separated by sodium dodecyl sulfate polyacrylamide gel electrophoresis (SDS-PAGE), followed by identification with liquid chromatography-mass spectrometry (LC–MS). Protein extraction involved the dissolution of substrates in either buffer-1 (Tris 100 mmol/L, SDS 1%, pH = 7.4) or buffer-2 (buffer-1, 1.4-dithiothreitol 1%). Freeze-dried AD and chitin-reduced AD required buffer-2, while the other substrates were mixed with buffer-1. To facilitate dissolution, the samples were sonicated (3 × 6 pulses, power 60% for 2 s, HTUSONI130, G. Heinemann, Germany). Subsequently, the proteins in the samples were precipitated with acetone (freeze-dried TM and chitin-reduced TM) or methanol (all other substrates). After centrifugation (17′949 x g, 4°C, 10 min), the supernatants were discarded, and the pellets were mixed with acetone or methanol once again. If necessary (i.e., freeze-dried AD), the samples were sonicated (2 × 6 pulses, power 60% for 2 s). As the fat content in the samples was high, the centrifugation cycle was repeated to remove fat (at least two centrifugation steps were performed for all samples). After the last centrifugation step, the supernatants were discarded, the pellets were resuspended in buffer-1, the proteins were allowed to solubilize at 20°C for 1 hour, the samples were sonicated (1 × 6 pulses), centrifuged, and the protein-containing supernatants were collected. The mass spectrometry proteomics data have been deposited to the ProteomeXchange Consortium via the PRIDE partner repository with the dataset identifier PXD041521 (52, 53).

The concentration of proteins in the samples was analyzed using the bicinchoninic acid assay, as introduced in Smith et al. (54). For SDS-PAGE analysis, the samples were diluted with a 6x sample buffer (Tris–HCl 350 mmol/L, glycerol 50%, SDS 10%, 1.4-dithiothreitol 100 mmol/L, pH = 6.8). Prior to gel electrophoresis, the proteins were denatured at 95°C for 5 min. Samples of equal volume and protein concentrations, together with a molecular weight marker (Benchmark, Invitrogen, United States), were separated by SDS-PAGE (polyacrylamide 15%). The gels were stained with colloidal Coomassie according to the procedure of Kang et al. (55).

Subsequently, peptide mass fingerprinting was performed as described elsewhere (56, 57). Briefly, the polyacrylamide gel pieces were manually excised from the protein bands and washed three times, alternating between a 100 μL destaining buffer (ammonium bicarbonate 25 mmol/L, acetonitrile 50% v/v) and a 100 μL digestion buffer (ammonium bicarbonate 25 mmol/L). Tryptic-in-gel digestion of proteins was performed with 2 μL trypsin (4 mg/L) in 20 μL of digestion buffer at 25°C for 48 h. The peptides were then separated on an XTerra MS C18 column (3.5 μm, 1.0 mm x 150 mm, Waters, United States) by a Rheos 2,200 HPLC (Flux Instruments AG, Switzerland) hyphenated to a linear ion trap mass spectrometer (LTQ, Thermo Scientific, Switzerland) with an electron spray ionization interface. To identify parental proteins, fragmentation data were matched to Uniprot databases (March 2020) by the Mascot search engine (Matrix Science, United Kingdom), with the search settings: database: Swissprot or Trembl; enzyme: trypsin; maximum miscleavages: 1; peptide and MS/MS tolerance: 0.8; variable modifications: deamidated (NQ), Gln- > pyro-GLU (N-term Q), oxidation (M); significance threshold: *p* < 0.05; ions score cutoff: 20. The identification results were manually validated based on the protein (≥ 50) and peptide score (≥ 25), with at least three different peptides and protein sequence coverage (> 10%). Original Mascot dat files were uploaded to the PRIDE proteomics identifications database (PXD041373) (52, 53).

### 2.4. *In vitro* digestion (IVD)

IVD was performed using the static INFOGEST protocol (18, 19) with the substrates listed in Table 1. As recommended by Brodkorb et al. (18), the activities of digestive enzymes and concentrations of bile salts were determined with the corresponding assays, and pH-test adjustment experiments were conducted for all substrates to account for individual buffer capacities. The substrates were ground into smaller pieces with a food processor (Moulinex DPA3, Moulinex, France) to simulate the effect of mastication. This step was omitted for substrates that were already in powder form due to processing or food preparation. The amount of substrate corresponding to 40 mg of protein was mixed with water to yield 1 gram of food. The digestion of 1 gram of a protein-free cookie was carried out in parallel to the test foods to provide background measurements with minimal autolysis of digestive enzymes (58). The cookie was prepared as reported in Sousa et al. (59). For subsequent IVD, the protocol of Minekus et al. (19) was followed. The oral phase was conducted without amylase because of the lack of starch in the substrates. Moreover, some adjustments to improve the homogeneity of the pancreatin suspension for the intestinal phase were implemented, as described in Sousa et al. (20): Pancreatin was mixed with SIF according to the protocol and then vortexed for 10 s. After ultrasonic treatment for five minutes, the mixture was centrifuged (2000 x g, 4°C, 5 min) and the supernatant immediately used for the intestinal phase. In the oral phase, the samples were diluted (1:1 wt/wt) with simulated salivary fluid (pH = 7, 37°C). The oral bolus was then mixed (1:1 vol/vol) with simulated gastric fluid (pH = 3, 37°C) containing pepsin (2000 U/mL) and incubated at 37°C on a rotating wheel for two hours. The intestinal phase was started with the dilution (1:1 vol/vol) of the gastric chyme with simulated intestinal fluid (pH = 7, 37°C) containing pancreatin (at 100 U trypsin activity/mL) and bile (10 mmol/L). After a two-hour incubation time at 37°C on a rotating wheel (Stuart™ Rotator SB3, Bibby Scientific^™^, United Kingdom), digestion was stopped by a protease inhibitor (1 mmol/L; AEBSF, trademark Pefabloc^®^, 500 mmol/L, Roche, Switzerland). Upon sampling, the digested samples were immediately snap frozen in liquid nitrogen (18). At least three individual experiments per substrate were conducted to assess the total protein *in vitro* digestibility and *in vitro* digestibility of individual AA. Moreover, the kinetics of IVD with 10 gastric and 10 gastrointestinal sampling times were carried out for peptide analysis (amino acid counting), in which the pepsin activity in the gastric phase was stopped by a pH adjustment to 7.

### 2.5. Amino acid counting (LC–MS)

For analysis of the generated peptides during IVD, digested samples were split into supernatants and pellets by centrifugation (13′000 x g, 4°C, 15 min). The supernatants were filtered through Amicon columns (Ultracel YM-30, Millipore, Switzerland) and the peptides analyzed by a Rheos 2,200 HPLC (Flux Instruments AG, Switzerland), which was equipped with a XTerra MS C18 column (3.5 μm, 1.0 mm x 150 mm, Waters, United States) and hyphenated to a linear ion trap mass spectrometer (LTQ, Thermo Scientific, Switzerland). The peptides were measured in three overlapping narrow-mass windows spanning between 100 and 1,300 m/z. The three raw files were merged and submitted to Mascot (Matrix Science, United Kingdom) for an identification search within a database containing a subset of the previously identified proteins from TM, AD, and chicken. The criteria for protein selection included identification in all substrates of the same species and a high intensity of band on SDS-PAGE. The peptides of the same protein were aligned with the protein sequence and the AA, identified within a peptide, were summed up and mapped along the protein sequence, colored according to their abundance as previously described (56).

### 2.6. Amino acid analysis of digesta (TAA method)

The total protein *in vitro* digestibility and *in vitro* digestibility of individual AA was assessed at the end of the IVD. The detailed procedure was published recently (20). Firstly, the digested substrates and cookie were split into digestible (potential absorbable, consisting of free AA and peptides up to 8 to 10 AA) and indigestible (non-absorbable) fractions by precipitation of the proteins and larger peptides with methanol (80% vol/vol, final concentration) at −20°C for 1 hour and subsequent centrifugation (2′000 x g, 4°C, 10 min). The supernatants (digestible fraction) were collected. The pellets (indigestible fraction) were washed twice with methanol (100%), centrifuged (2′000 x g, 4°C, 5 min), and dried with a CentriVap (Labconco, United Kingdom). The weights of the supernatants and pellets were noted accurately throughout the procedure for the final digestibility calculations. Secondly, the pellets and supernatants were hydrolyzed with hydrochloric acid 6 mol/L for 15 h at 110°C. In short, 220 μL of supernatants were dried in a CentriVap and resuspended in 220 μL milli Q water, 120 μL 3.3′-dithiodipropionic acid 0.1% / NaOH 0.2 mol/L, 120 μL HCl 0.2 mol/L, 40 μL Norvalin 10 mmol/L, and HCl 37%. The dried pellets were hydrolyzed in four times the hydrolysis volume of the supernatants. The hydrolysis mixtures were flushed with nitrogen gas before incubation to minimize oxidation. Thirdly, TAA analysis was performed according to the AOAC method 2018.06 for infant formula (60), with modifications described in Sousa et al. (20).

### 2.7. Quantification of primary amines in digesta (R-NH_2_ method)

As an alternative to the TAA method, total protein *in vitro* digestibility was determined by quantifying the primary amines in the digestible and indigestible fractions of the digested substrates and cookie (20). The same procedure as described for the TAA method, i.e., separation of digestible and indigestible fractions followed by acid hydrolysis, was conducted. The hydrolyzed samples were diluted five (supernatants) and ten (pellets) times with perchloric acid 0.5 mol/L to precipitate the proteins and longer peptides. After derivatization with o-phthaldialdehyde, the produced 1-alkylthio-2-alcylisonindol compound was measured on a UV/VIS spectrophotometer at 340 nm. Free amino acids and small peptides in the samples were quantified based on a glutamic acid standard curve (56, 61).

### 2.8. Calculation of *in vitro* digestibility, *in vitro* DIAAR, and proxy *in vitro* DIAAR

The total amount of individual AA (mg; TAA method) and primary amines (mmol glutamic acid equivalents; R-NH_2_ method) were quantified for the hydrolyzed digestible (supernatants) and indigestible (pellets) fractions of the *in vitro* digested substrates and protein-free cookie, considering the weight of each fraction and dilution steps during the analysis. To account for the enzyme background from the IVD, the total amount of AA or the total amount of primary amines in the cookie fractions (Cookie supernatant = Cs, Cookie pellet = Cp) were subtracted from the total amount in the corresponding substrate fractions (Food supernatant = Fs, Food pellet = Fp). The total amount in the pellet of a highly digestible substrate is similar to the pellet of the cookie (both containing the same digestive enzymes background), and due to analytical variability, the subtraction (Fp-Cp) might result in negative values, which is why the minimal value for (Fp-Cp) was set to zero.

Both the *in vitro* digestibility of individual AA (TAA method) and the total protein *in vitro* digestibility (R-NH_2_ method) were calculated by dividing the corrected digestible fraction by the corrected total of digestible and indigestible fractions based on Eq. 1 (20):


(1) $$invitro\;\mathrm{digestibility}\;\left[\%\right]=\frac{\left(Fs-Cs\right)\;}{\left(\left(Fs-Cs\right)+\max\left(0;Fp-Cp\right)\right)}\times 100\;$$

With Eq. 1, the *in vitro* digestibility of individual AA could be assessed using the results from the TAA method, with the mean digestibility of all individual AA resulting in the total protein *in vitro* digestibility. In contrast, by measuring the primary amines (R-NH_2_ method), only the total protein *in vitro* digestibility could be derived, without any distinction between the digestibility of individual AA.

The *in vitro* digestible IAA content (DIAA) for each IAA in one gram of food protein (TN x 6.25) was calculated with equation (20).


(2) $$\begin{array}{l} invitroDIAA=mg\;of\;IAA\;per\;g\;offoodprotein\\ \;\;\;\;\;\;\;\;\;\;\;\;\;\;\;\;\;\;\;\;\;\;\;\;\;\;\;\;\;\;\;\;\;\;\;\;\;\;\;\times invitrodigestibilityof\;IAA \end{array}$$

The *in vitro* digestible indispensable amino acid (reference) ratio (DIAAR) was calculated as recommended by FAO (17), based on Eq. 3, by dividing the *in vitro* DIAA by the AA pattern of a reference protein, which reflects the dietary IAA requirements for either 1) infants (birth to 6 months), 2) young children (6 months to 3 years), or 3) older children, adolescents, and adults (17). For a given reference pattern, the reported *in vitro* DIAAS of a food is the lowest of the nine calculated DIAAR, and the IAA with the lowest value is considered the first limiting IAA. For legal purposes, FAO (17) requests the use of the reference pattern for young children.


(3) $$invitroDIAAR\;\left[\%\right]=\frac{\begin{array}{l} mg\;ofin\;\;vitroDIAA\\ in\;1\;g\;ofdietaryprotein\; \end{array}}{\begin{array}{l} \mathrm{mg}\;\mathrm{of the same dietary}\;\mathrm{IAA}\;\\ \mathrm{in}\;1\;g\;\mathrm{of the reference protein} \end{array}}\times 100$$

An approximation of DIAAR (named: proxy *in vitro* DIAAR) was calculated based on Eqs 4, 5, where the *in vitro* digestibility of individual IAA was replaced by the total protein *in vitro* digestibility obtained by the TAA or R-NH_2_ method, as introduced by Sousa et al. (20).


(4) $$\begin{array}{l} proxyinvitroDIAA=mg\;of\;IAA\;per\;g\;foodprotein\\ \;\;\;\;\;\;\;\;\;\;\;\;\;\;\;\;\;\;\;\;\;\;\;\;\;\;\;\;\;\;\;\;\;\;\;\;\;\;\;\;\;\;\;\;\;\;\;\;\;\;\;\;\;\times totalinvitrodigestibility \end{array}$$

(5) $$proxyin\;vitroDIAAR=\frac{\begin{array}{l} mg\;ofproxyin\;vitroDIAA\\ in\;1\;g\;ofdietaryprotein \end{array}}{\begin{array}{l} mg\;ofthesamedietary\;IAA\;\\ in\;1\;g\;ofthereferenceprotein \end{array}}\times 100$$

Moreover, the *in vitro* DIAAR and proxy *in vitro* DIAAR were determined by using the protein content calculated by the sum of anhydrous AA instead of crude protein (TN x 6.25), which is required for Eqs 2, 4.

### 2.9. Statistical analysis

The results are presented as mean ± standard deviations (SD). The data analyses were performed with Microsoft Excel and R and the statistical analyses with IBM SPSS Statistics 28.0. The statistical differences in macronutrients, total protein *in vitro* digestibility, *in vitro* DIAAR, and *in vitro* DIAAS between the substrates were analyzed by one-way analysis of variance (ANOVA), using Bonferroni’s test as a post-hoc test. The level of significance was set at *p* < 0.05.

## 3. Results

### 3.1. Nutrient composition

The nutrient composition of the substrates are presented in Table 2. In comparison with chicken, regardless of the processing, TM and AD had considerably higher fat contents (*p* < 0.001). Crude fat was lower in AD than TM when comparing similar processing methods (*p* < 0.001), while it was highest in the chitin-reduced substrates compared to the other processing methods in both species (*p* < 0.001). The crude protein content of TM and AD was high at 34.2 to 56.6% and 39.1 to 70.0% in the dry matter (DM), respectively, with the lowest values found in the chitin-reduced substrates (*p* < 0.05). The chitin content of TM and AD were low at 4 resp. 3% DM, whereas the chitin-reduced counterparts were virtually free of chitin.

**Table 2:** Characterization of substrates subjected to *in vitro* digestion.

	TM blanched	TM freeze-dried/pulverized^1^	TM chitin-reduced	AD blanched	AD oven-dried	AD freeze-dried/pulverized^1^	AD chitin-reduced	Chicken breast
DM	37.61 (±1.47)^a^	96.30 (±0.87)^bc^	97.30 (±0.01)^c^	25.69 (±0.57)^d^	90.39 (±0.25)^e^	94.51 (±0.02)^bc^	93.48 (±0.11)^be^	28.36 (±0.00)^d^
Fat	30.75 (±0.51)^a^	35.53 (±0.29)^b^	46.89 (±0.48)^c^	21.56 (±0.17)^d^	26.56 (±0.17)^e^	28.16 (±0.23)^e^	34.38 (±0.63)^b^	5.96 (±0.10)^f^
Protein (TN x 6.25)	52.8 (±2.9)^a^	56.6 (±1.6)^ab^	34.2 (±0.1)^c^	70.0 (±5.5)^d^	65.1 (±3.4)^bd^	64.2 (±1.1)^abd^	39.3 (±0.2)^c^	94.4 (±0.2)^e^
Chitin^2^	n.d.	4.00 (±0.3)	< 0.07	n.d.	n.d.	3.2 (±0.2)	< 0.07	n.d.
*Indispensable AA (IAA)*								
HIS	1.8 (±0.1)	1.7 (±0.0)	1.1 (±0.0)	1.7 (±0.1)	1.5 (±0.0)	1.5 (±0.1)	0.7 (±0.0)	2.9 (±0.1)
ILE	2.6 (±0.2)	2.4 (±0.0)	1.3 (±0.0)	2.9 (±0.1)	2.5 (±0.1)	2.6 (±0.0)	1.1 (±0.0)	4.8 (±0.1)
LEU	4.2 (±0.1)	3.9 (±0.1)	1.9 (±0.0)	4.9 (±0.2)	4.2 (±0.4)	4.4 (±0.2)	1.7 (±0.1)	7.3 (±0.2)
LYS	3.2 (±0.2)	3.0 (±0.0)	1.9 (±0.2)	4.2 (±0.2)	3.3 (±0.1)	3.6 (±0.1)	1.8 (±0.1)	8.4 (±0.3)
MET	0.8 (±0.0)	0.7 (±0.0)	0.4 (±0.0)	1.1 (±0.0)	0.9 (±0.2)	1.1 (±0.0)	0.4 (±0.0)	2.8 (±0.0)
PHE	2.2 (±0.1)	2.0 (±0.1)	1.2 (±0.1)	2.5 (±0.1)	2.1 (±0.1)	2.4 (±0.0)	0.9 (±0.0)	3.7 (±0.1)
THR	2.3 (±0.1)	2.1 (±0.0)	1.1 (±0.0)	2.7 (±0.1)	2.2 (±0.1)	2.4 (±0.0)	1.2 (±0.0)	4.2 (±0.1)
TRP	0.6 (±0.0)	0.6 (±0.0)	0.5 (±0.0)	0.6 (±0.0)	0.6 (±0.0)	0.6 (±0.0)	0.3 (±0.0)	1.2 (±0.0)
VAL	3.8 (±0.1)	3.5 (±0.1)	1.7 (±0.0)	4.2 (±0.2)	3.6 (±0.2)	3.8 (±0.1)	1.7 (±0.0)	5.1 (±0.1)
*Dispensable AA (DAA)*								
ALA	4.2 (±0.2)	3.9 (±0.1)	1.5 (±0.0)	6.0 (±0.2)	5.1 (±0.8)	5.0 (±0.2)	2.7 (±0.1)	5.2 (±0.1)
ARG	3.1 (±0.2)	2.9 (±0.0)	1.9 (±0.0)	4.6 (±0.2)	3.9 (±0.3)	3.9 (±0.1)	2.6 (±0.0)	6.1 (±0.1)
ASP	4.2 (±0.0)	3.9 (±0.4)	2.7 (±0.2)	5.0 (±0.2)	4.6 (±1.0)	4.5 (±0.4)	2.4 (±0.1)	8.0 (±0.2)
CYS	0.6 (±0.0)	0.6 (±0.0)	0.4 (±0.0)	0.8 (±0.0)	0.7 (±0.0)	0.7 (±0.0)	0.5 (±0.0)	1.0 (±0.1)
GLU	6.0 (±0.1)	5.6 (±0.3)	3.5 (±0.2)	7.2 (±0.3)	6.2 (±0.4)	6.1 (±0.4)	4.2 (±0.0)	11.9 (±0.2)
GLY	3.1 (±0.1)	2.9 (±0.1)	1.3 (±0.0)	3.8 (±0.2)	3.3 (±0.4)	3.2 (±0.1)	2.2 (±0.0)	3.9 (±0.1)
PRO	3.8 (±0.1)	3.5 (±0.2)	4.4 (±0.1)	4.1 (±0.1)	3.5 (±0.2)	3.5 (±0.1)	2.0 (±0.1)	3.3 (±0.1)
SER	2.6 (±0.1)	2.4 (±0.1)	1.2 (±0.0)	2.9 (±0.1)	2.6 (±0.4)	2.5 (±0.1)	1.2 (±0.0)	3.5 (±0.1)
TYR	3.0 (±0.9)	3.4 (±0.7)	1.9 (±0.1)	2.6 (±0.1)	2.7 (±0.5)	3.6 (±0.7)	1.2 (±0.0)	2.2 (±0.1)
Total AA^3^	44.46 (±0.89)	42.01 (±0.82)	25.73 (±0.29)	52.67 (±0.53)	45.63 (±1.45)	47.45 (±0.82)	24.63 (±0.16)	73.30 (±0.49)
Molar ratio (IAA/DAA)	0.61	0.60	0.53	0.58	0.56	0.60	0.45	0.80

1 Pulverized substrates are assumed to have the same nutrient composition as freeze-dried substrates.

2 The LOD and LOQ of the method are 2.4 and 7.1 μg/mL, respectively, calculated with SD regression.

3 Protein content based on the summation of anhydrous amino acids.

Values are means ± SDs in g/100 g dry substrate. Values without common letter denotes significant differences (*p* < 0.05) between the substrates. Individual AA were calculated with molar mass of respective AA, and total AA is the summation of all AA calculated with molar mass of anhydrous AA (molar mass of AA minus molar mass of water). n.d. = not determined, AD = *A. domesticus*, TM = *T. molitor* larvae.

Chicken protein was characterized by a molar ratio of indispensable/dispensable AA (IAA/DAA) of 0.80. Interestingly, all TM and AD substrates were very similar in AA composition, yielding an IAA/DAA molar ratio of = 0.6, except for the chitin-reduced substrates, with a slightly lower molar ratio of 0.5. The most abundant AA for all substrates was glutamic acid, apart from the chitin-reduced TM, for which it was proline. Leucine was the most abundant IAA for the blanched TM, freeze-dried TM, chitin-reduced TM, blanched AD, oven-dried AD, and freeze-dried AD. Lysine was the most abundant IAA for the chitin-reduced AD and chicken. The least abundant AA was tryptophan in all AD substrates and cysteine in all TM substrates and chicken. Tryptophan was the least abundant IAA for all substrates.

### 3.2. Protein identification

**Figure 1:**
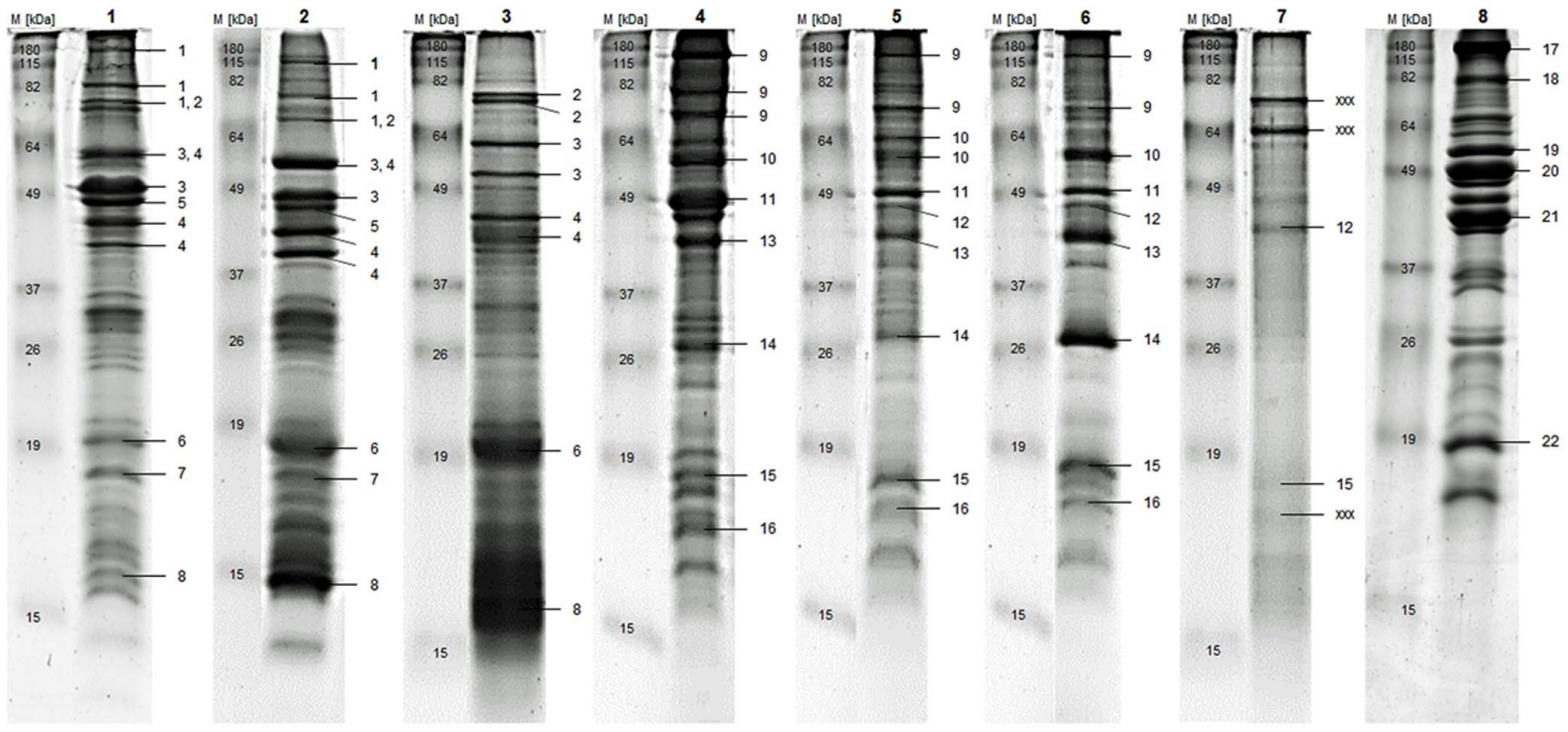
Gel bands are labeled by one or multiple numbers to indicate identified proteins, which are listed according to their numbers in Table 3. M) Standard Protein Marker, 1) blanched TM, 2) freeze-dried TM, 3) chitin-reduced TM, 4) blanched AD, 5) oven-dried AD, 6) freeze-dried AD, 7) chitin-reduced AD, 8) chicken; AD = *A. domesticus*, TM = *T. molitor* larvae.

In Figure 1 and Table 3, identification of the main proteins of each substrate are presented, while the detailed protein identifications are shown in the Supplemental material (Supplementary Figures S1A,B and Supplementary Table S1). In all substrates, including chicken, numerous muscle proteins, such as *Myosin heavy Chain* (Figure 1: No. 1, 9, and 17), *Actin* (Figure 1: No. 5, 11, and 20) and *Tropomyosin* (Figure 1: No. 4, 12, 13, and 21) were identified. The chicken additionally contained proteins that are part of the glycolysis pathway (e.g., *Beta-Enolase*, Figure 1: No. 19; and *Glyceraldehyde-3-phosphate Dehydrogenase*, Supplementary Figure S1A: No. 48), many additional muscle proteins, such as *Troponin I* (Supplementary Figure S1A: No. 57) and *Myosin regulatory light Chain 2* (Figure 1: No. 22), and proteins that play a role in cellular energy homeostasis (e.g., *Adenylate Kinase Isoenzyme 1*, Supplementary Figure S1A: No. 54). TM and AD were comparable to chicken when considering that many proteins were related to muscle function, but they clearly differed by species-specific proteins.

**Table 3:** Proteins identified by peptide mass fingerprinting after tryptic-in-gel digestion.

	Mass [Da]	Protein description
*Tenebrio molitor larvae* (TM)		
1	224,465	Myosin heavy chain, muscle
2	90,623	86 kDa early-staged encapsulation-inducing protein
3	62,445	56 kDa early-staged encapsulation-inducing protein
4	32,428	Tropomyosin
5	41,816	Actin
6	65,481	Cockroach allergen-like protein
7	17,027	Myosin light chain alkali-like protein
8	14,138	12 kDa Hemolymph protein b
*Acheta domesticus* (AD)		
9	224,465	Myosin heavy chain
10	46,708	Troponin T
11	41,785	Actin, muscle
12	23,039	Tropomyosin 2 (fragment)
13	23,014	Tropomyosin 1 (fragment)
14	22,594	Myosin light chain
15	19,803	Apolipophorin-III
16	13,829	Histone H2B
*Chicken breast*		
17	223,145	Myosin heavy Chain, skeletal muscle, adult
18	104,275	Alpha-Actinin-2
19	47,196	Beta-Enolase
20	42,051	Actin, alpha skeletal muscle
21	32,765	Tropomyosin alpha-1 chain
22	18,839	Myosin regulatory light chain 2, skeletal muscle isoform
xxx	–	no identifications

The major proteins found exclusively in TM were *86 kDa* and *56 kDa early-staged Encapsulation proteins* (Figure 1: No. 2 and 3), which are thought to be components of the insect cellular defense reaction (62). Furthermore, *Cockroach Allergen-like Protein* (Figure 1: No. 6), various hemolymph proteins (e.g., *28 kDa Desiccation Stress Protein*, Supplementary Figure S1A: No. 11), and *12 kDa Hemolymph Protein b* (Figure 1: No. 8), as well as proteolytic (*putative serine-* and *putative trypsin-like Proteinases*, Supplementary Figure S1A: No. 10 and 13) and catalytic (*Alpha-Amylase* and *Chitinase*; Supplementary Figure S1A: No. 4 and 9) enzymes were identified in TM. The specific proteins for AD are associated with a wide range of functions: stress response (*Heat Shock 70 kDa Protein Cognate 4*, Supplementary Figure S1A: No. 24), structural components of microtubules (*Tubulin beta-1 and alpha-1 Chains*, Supplementary Figure S1B: No. 26 and 27), lipid transport during insect flight (*Apolipophorin-III*, Figure 1: No. 15), core components of nucleosome (*Histone H2B* and *Histone H4*, Supplementary Figure S1B: No. 37 and 39), and ATP-Phosphotransferase (*Arginine Kinase*, Supplementary Figure S1B: No. 29).

The substrates from the same species with different food processing had the most proteins in common, especially the main and most abundant proteins. However, by comparing the raw to blanched and freeze-dried to chitin-reduced substrates, the differences were assessed in gel band patterns and total identification results. The bands of the chitin-reduced TM were less intense and had fewer proteins compared to the other TM substrates. This was even more pronounced with the chitin-reduced AD, showing almost no bands on the gel, of which hardly any proteins were identified with peptide mass fingerprinting.

### 3.3. Peptide identification during IVD

**Figure 2:**
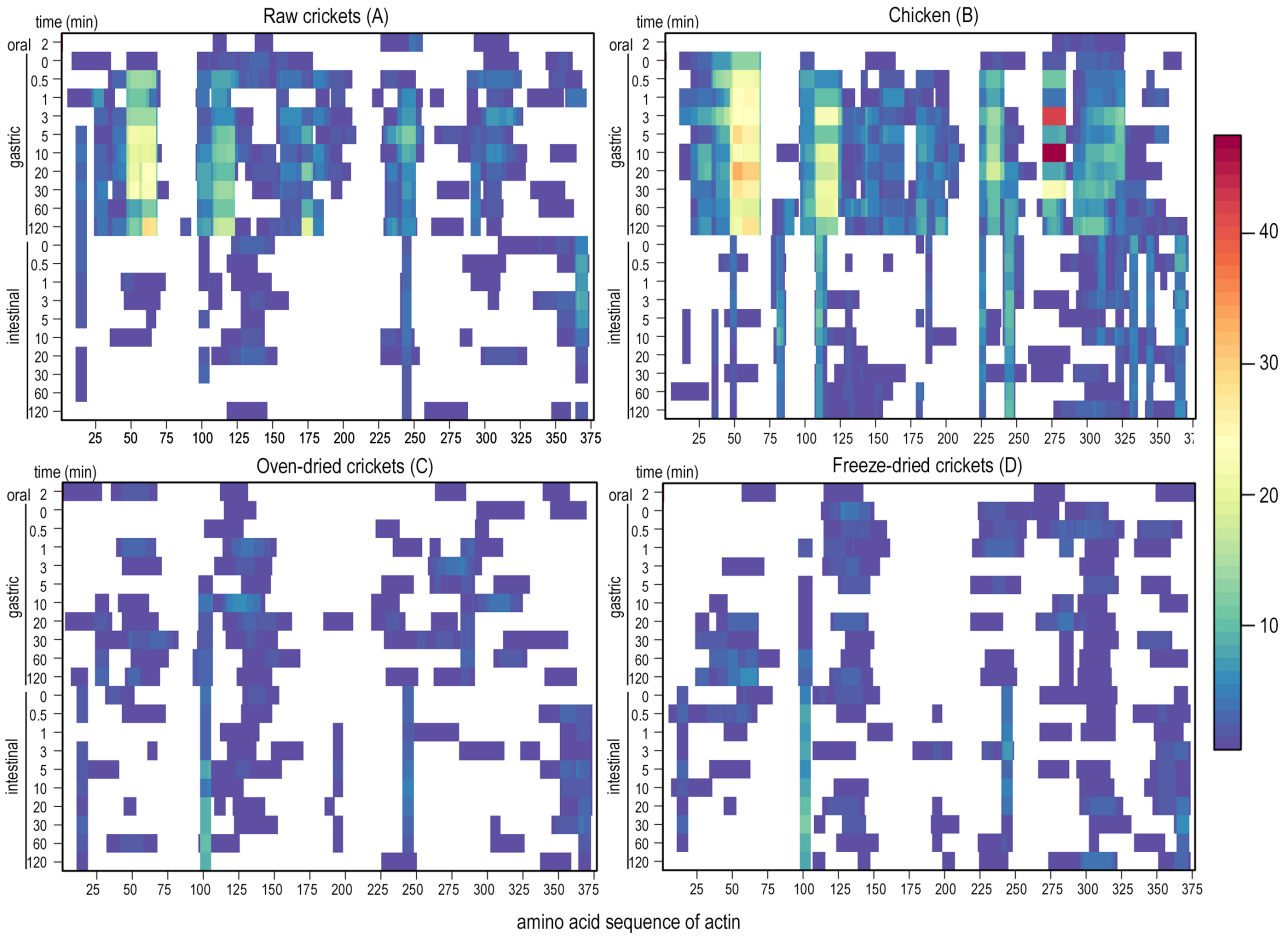
Peptides of *Actin* generated during IVD of **(A)** raw crickets, **(B)** chicken, **(C)** oven-dried crickets, and **(D)** freeze-dried crickets, with the protein sequence on the x-axis (E0VKP4_PEDHC and ACTS_CHICK for crickets and chicken, respectively), and the 21 different sampling times during *in vitro* digestion on the y-axis. Abundance of AA within identified peptides of the protein of interest is shown in color-code along the protein sequence, while the white regions indicate no peptide identifications.

No fully intact proteins of any of the substrates were detected at the end of the IVD, when the SDS-PAGE gels of digesta at the intestinal endpoint were analyzed by peptide mass fingerprinting (data not shown). The time-dependent release of peptides during IVD of a selection of major proteins was measured by sampling digesta at 21 different timepoints, and visualization by a heat map revealed variability in the hydrolysis of the same protein in different substrates, as well as between different proteins in the same substrate. Figure 2 compares the protein hydrolysis of *Actin* during IVD of raw AD (A), oven-dried AD (C), freeze-dried AD (D), and chicken (B), which showed a clear distinction in peptide patterns between the wet (raw AD and chicken) and the dried (oven-dried and freeze-dried AD) substrates, rather than a difference between species. Although the protein sequence of *Actin* identified in AD (E0VKP4_PEDHC) and chicken (ACTS_CHICK) were not identical, there was an identity of over 92%, corresponding to 350 identical positions of the total 375 positions in the sequence (63). While many peptides were identified throughout the gastric phase of raw AD and chicken in four distinct regions of the protein sequence (regions: 25–65, 95–210, 225–250, and 285–325), clearly fewer peptides were detected during the intestinal phase (Figure 2). This was not the case for the oven-dried AD and freeze-dried AD, however, as fewer peptides were generated in the gastric phase compared to the raw AD and chicken, and no clear distinction between the gastric and intestinal phase regarding the peptide number was discernible.

*Actin* was digested in a blockwise manner in all the analyzed substrates, leaving the in- between protein regions mostly undigested. These digestive enzyme-resistant regions of *Actin* were rather short in comparison to the peptide patterns of *Cockroach Allergen-like Protein*, which were measured during the IVD of the TM substrates, where few peptides were identified in the gastric and intestinal phases, with many regions without any or just a few identified peptides (Supplementary Figure S2A). In contrast to this, the *56 kDa early-staged Encapsulation* Protein, which is another protein of the TM substrates, generated many peptides in the gastric and intestinal phases in all substrates that fully covered the protein sequence (Supplementary Figure S2B). As the hydrolysis of individual proteins from the same substrate varied considerably, changes to the protein composition, resulting from the processing and food preparation steps, is likely to also affect total protein digestibility.

### 3.4. Total protein *in vitro* digestibility

**Figure 3:**
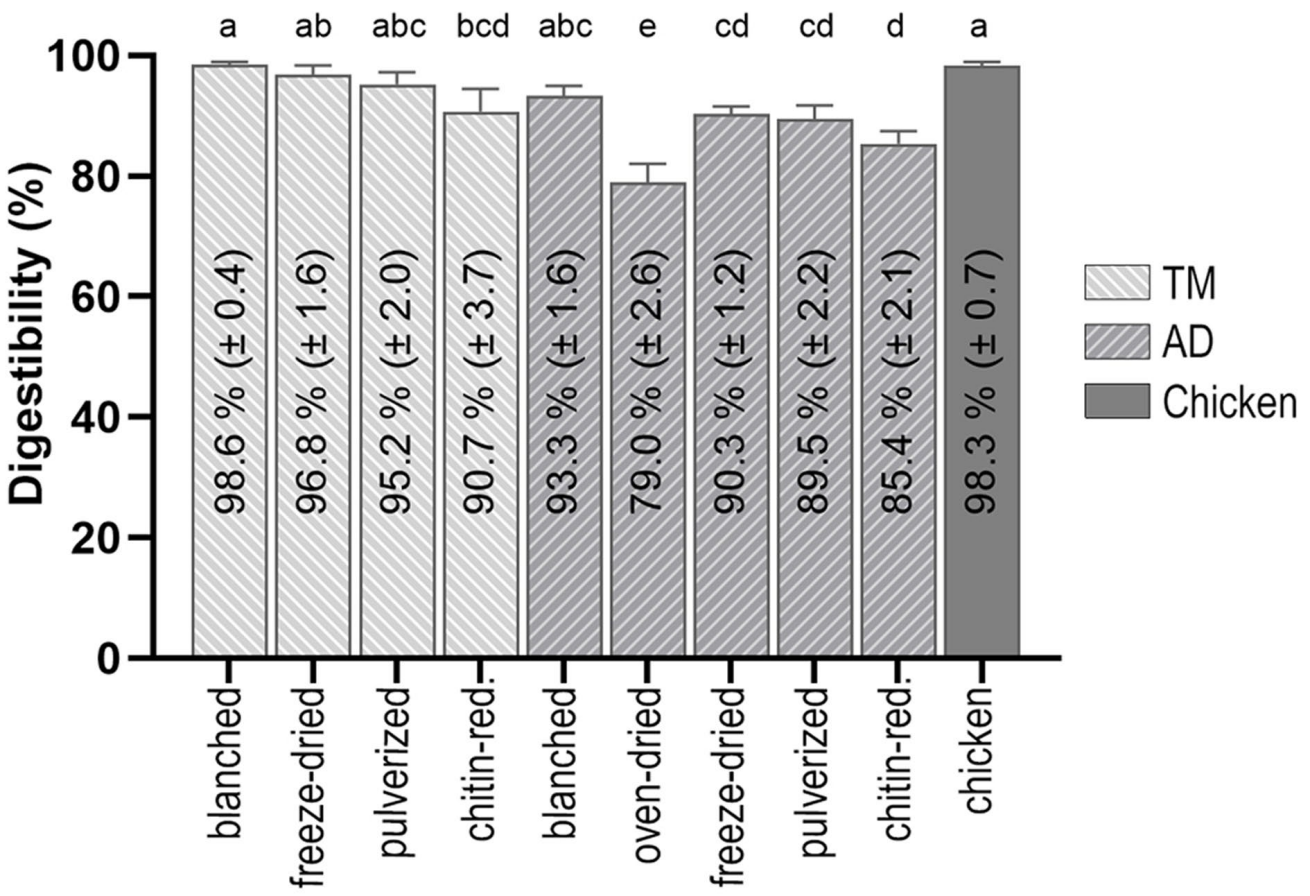
Total protein *in vitro* digestibility determined by TAA analysis of *T. molitor* larvae (TM) and *A. domesticus* (AD) undergoing different processing and food preparation, in comparison to cooked chicken breast. Bars without common letters differ (*p* < 0.05).

The total protein *in vitro* digestibility for the substrates ranged from 79.0 to 98.6%, as determined by the TAA analysis (Figure 3). Chicken had a high total protein *in vitro* digestibility, which was not statistically different from the blanched TM, freeze-dried TM, and blanched AD. Oven-drying and chitin-reduction negatively affected the total protein *in vitro* digestibility, with the overall lowest value observed for the oven-dried AD, in comparison to all the measured substrates (*p* < 0.05). In general, the AD substrates had slightly lower total protein *in vitro* digestibility values when compared to the TM substrates with an equal degree of processing, although these differences were not significant, except for the freeze-dried substrates (*p* < 0.05).

When comparing the TM substrates, no difference could be found between the total protein *in vitro* digestibility of the blanched TM, freeze-dried TM, and pulverized TM. The lowest value of all the TM substrates was found in the chitin-reduced TM, which was significantly lower than the blanched TM (*p* = 0.006). The highest total protein *in vitro* digestibility in the AD substrates was found for the blanched AD, freeze-dried AD, and pulverized AD, while the chitin-reduced AD was significantly less digestible than the blanched AD (*p* = 0.005) but more digestible than the oven-dried AD (p < 0.05).

TAA analysis (Figure 3) resulted in a slightly higher total protein *in vitro* digestibility than R-NH_2_ analysis (Supplementary Figure S3), but both measurements resulted in similar relative results. The advantage of TAA analysis is the possibility to determine the digestibility of individual AA, shown in Supplementary Table S1, which is required for the calculation of *in vitro* DIAAR. Generally, *in vitro* digestibility was the lowest for most AA in the chitin-reduced TM, chitin-reduced AD, and oven-dried AD and highest in either the chicken, blanched TM, or freeze-dried TM, therefore mirroring the pattern of total protein *in vitro* digestibility. The lowest values of *in vitro* digestibility of individual AA were observed for tyrosine, cysteine, and tryptophan, the latter being an IAA.

### 3.5. Protein quality measures: *in vitro* DIAAS and *in vitro* proxy *in vitro* DIAAS

**Figure 4:**
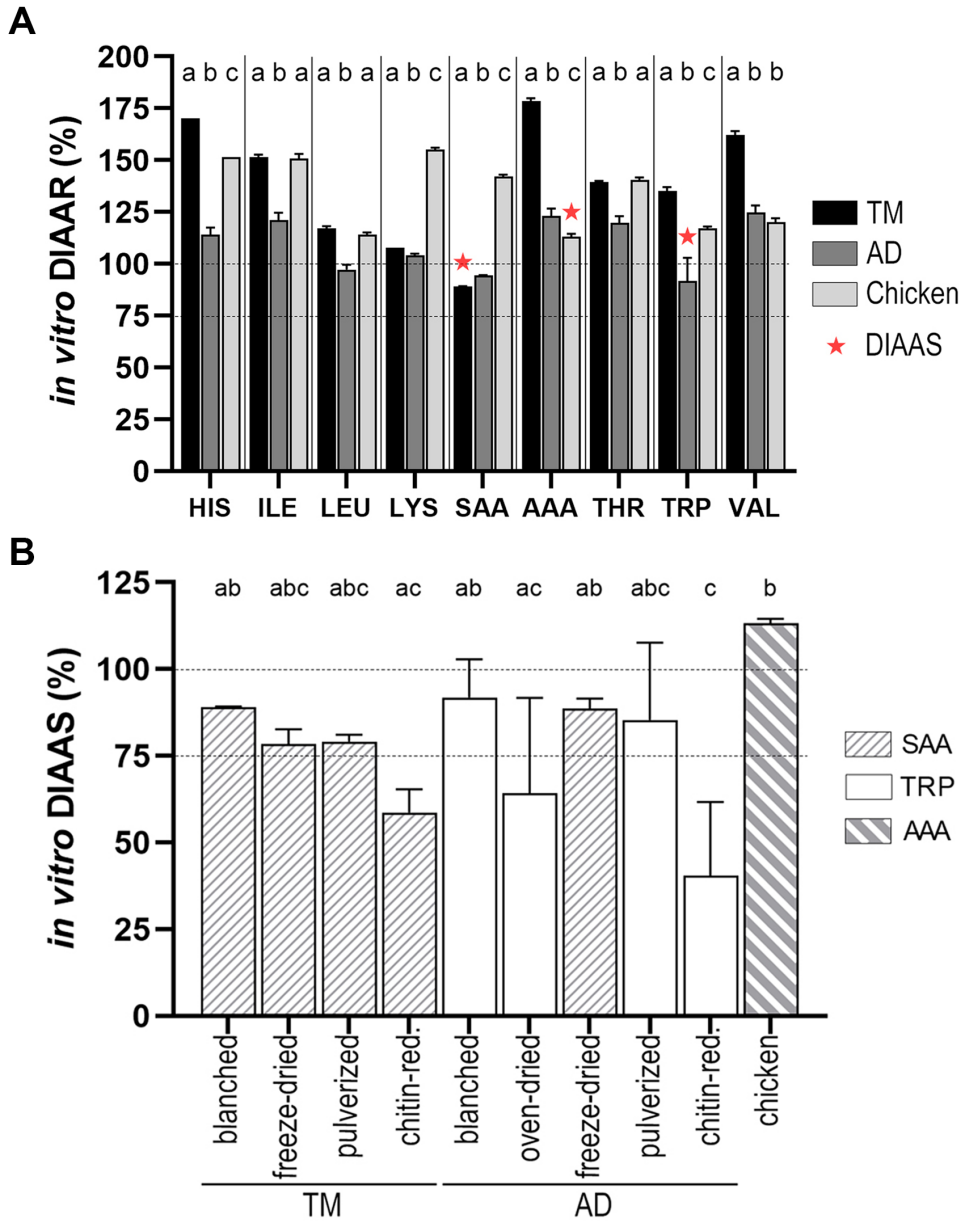
**(A)** Comparison of *in vitro* DIAAR for the AA requirements of preschool children (6 months to 3 years) of blanched TM, blanched AD, and chicken. First limiting amino acid ratio (*in vitro* DIAAS = lowest DIAAR) is highlighted with red asterix. For each individual amino acid the bars without common letters differ. *In vitro* DIAAR for histidine was highest for blanched TM (*p* < 0.001) and lowest for blanched AD (*p* < 0.001). The *in vitro* DIAAR values for isoleucine, leucine and threonine did not differ for blanched TM and chicken, but was significantly lower for the blanched AD (*p* < 0.001). Lysine value was highest for chicken (*p* < 0.001) and blanched TM had a higher value than blanched AD (*p* = 0.002). Values for sulfur-containing AA were lowest for blanched TM (*p* < 0.001) and highest for chicken (*p* < 0.001). Aromatic AA was highest for blanched TM (*p* < 0.001), whereas blanched AD had a higher value than chicken (*p* = 0.004). *In vitro* DIAAR for tryptophan was higher for blanched TM than chicken (*p* < 0.05) and lowest value was found for blanched AD (*p* < 0.01). Valine value was highest for blanched TM (*p* < 0.001), while values for chicken and blanched AD did not differ. **(B)** *In vitro* DIAAS values for TM and AD undergoing different processing and food preparation are shown in comparison to the *in vitro* DIAAS of chicken. Dotted line categorizes DIAAS of the quality of protein sources in <75 (no claim); 75–99 (good); and ≥ 100 (excellent). AD = *A. domesticus*, TM = *T. molitor* larvae, DIAAR = digestible indispensable amino acid ratio, AAA, aromatic amino acids (tyrosine + phenylalanine), SAA, sulfuric amino acids (cysteine + methionine).

The *in vitro* DIAARs of the substrates were established for the reference IAA requirements for young children (6 months to 3 years) as requested by FAO for legal purposes (17), based on the *in vitro* digestibility of individual IAA (Supplementary Table S2) and total IAA contents in the substrates (Table 2). Blanched TM and blanched AD were the most suitable candidates for comparison with chicken, as other substrates underwent drying methods. The nine *in vitro* DIAAR values for these three substrates are presented in Figure 4 (A); the lowest *in vitro* DIAAR for a substrate is called *in vitro* DIAAS, and the corresponding IAA is the substrate’s first limiting AA. *In vitro* DIAAS for blanched TM (89.1 ± 0.1) and blanched AD (91.8 ± 11.1) were both lower than *in vitro* DIAAS for chicken (113.3 ± 1.2), which was the highest compared to all the substrates studied (Figure 4B), with aromatic AA (AAA) being the first limiting AA.

For all TM substrates, the sulfur-containing AA (SAA) presented the first limiting AA (Figure 4B), with *in vitro* DIAAS values between 75 and 100, except for the lowest score found in the chitin-reduced TM (58.7 ± 6.7). *In vitro* DIAAS for blanched TM (89.1 ± 0.1) was higher than pulverized TM (79.1 ± 2.0) and freeze-dried TM (78.4 ± 4.3). The first limiting AA for most AD substrates was tryptophan, with the highest *in vitro* DIAAS for blanched AD (91.8 ± 11.1), and the lowest *in vitro* DIAAS found in the chitin-reduced AD (40.4 ± 21.3). In contrast to the total protein *in vitro* digestibility, *in vitro* DIAAS of the oven-dried AD (64.2 ± 27.5) did not differ from the blanched AD.

These above presented results were calculated based on the *in vitro* digestibility of individual IAA, which results in the most accurate determination of *in vitro* DIAAR and *in vitro* DIAAS. In case only the total protein *in vitro* digestibility is available, e.g., R-NH_2_ analysis, an approximation of DIAAR (proxy *in vitro* DIAAR) may be determined. The *in vitro* DIAAS and proxy *in vitro* DIAAS values for additional reference amino acid scoring patterns (children younger than 6 months, and children older than 3 years), additional analytical methods (R-NH_2_ vs. TAA analysis), and calculations (crude protein as TN x 6.25 vs. protein content based on the summation of anhydrous AA) are summarized in the Supplementary material (Supplementary Table S3). Interestingly, the proxy *in vitro* DIAAS of R-NH_2_ analysis gave similar results as applying the mean *in vitro* digestibility of individual AA, assessed by TAA analysis. Overall, a slight overestimation occurred by calculating proxy *in vitro* DIAAS instead of *in vitro* DIAAS (Supplementary Table S2).

## 4. Discussion

### 4.1. Key results

Our main findings are that 1) *T. molitor larvae* (TM) and *A. domesticus* (AD), undergoing different processing and food preparation methods, are mostly complete proteins, providing adequate amounts of all IAA; 2) Total protein *in vitro* digestibility did not differ between chicken and the blanched insects, but it was negatively affected by oven-drying and, to a lesser extent, by chitin-reduction; 3) Processing and food preparation also affected the *in vitro* DIAAS values, which was highest for chicken (113) and ranged between 59 and 89 for TM and between 40 and 92 for AD, when considering the IAA requirements of young children aged 6 months to 3 years (17). The lowest *in vitro* DIAAS values were found in chitin-reduced and oven-dried substrates, highlighting the important role of identifying and optimizing suitable processing methods to maintain the quality of insect proteins for human consumption.

### 4.2. Nutritional relevance

Protein quality can be described as excellent if the DIAAS ≥100 and as good if the DIAAS is between 75 and 99, whereas no nutrition claim regarding protein quality should be made when the DIAAS <75 (17). Using these cutoff values, almost all the studied TM and AD qualify as a good protein source for children aged between 6 months and 3 years, except for chitin-reduced TM, chitin-reduced AD, and oven-dried AD with values below 75. Thus, blanched insects can be considered an excellent source of protein for children older than 3 years. Our findings are generally comparable to the reported *in vivo* DIAAS of 76 (SAA) for AD determined in growing pigs (27), and the *in vivo* DIAAS of 54 (SAA) for TM, which is within the range of our *in vitro* DIAAS values for the TM substrates. However, a direct comparison between our study and the study of Malla et al. is complicated, as the drying method as well as the processing steps, which can strongly affect the DIAAS, were not clearly described in the commercially obtained insects reported (27).

Chicken reached the highest *in vitro* DIAAS in our study, confirming it to be an excellent protein source for both young children (6 months to 3 years) and children older than 3 years. Our *in vitro* DIAAS for chicken was similar to the *in vivo* DIAAS values reported for other animal-based foods: 126 (Val) for conventionally-cured ham (34), 125 (His) for whey protein isolate (64), 122 (SAA) for egg (65), and 99 (Leu) for cooked ground beef (34), based on the standardized ileal AA digestibility in growing pigs and the IAA requirements of children older than 3 years. Plant-based foods have more variable protein quality scores: Legume-based foods can be good-quality proteins, such as cooked kidney beans (DIAAS of 74 (SAA) (15, 66)) and tofu (DIAAS of 97 (SAA) (67)), whereas cereals are a rather low protein-quality food group (68, 69). Thus, the protein quality of our studied insect species could be placed in the lower half of the animal-based foods or with the higher-quality plant-based foods, provided that a suitable food preparation method is applied. It is important to note that edible insects will be consumed in a mixed meal rather than as a single ingredient food, and balanced IAA profiles can be achieved by complementation of dietary protein sources. Considering the first limiting AA of TM and AD, complementation with foods that are rich in SAA and tryptophan, such as soybeans, nuts, and seeds (70), may increase the overall meal protein quality, and edible insects can, in turn, improve the protein quality of cereal proteins that are deficient in lysine.

The *in vitro* DIAAS values were calculated using crude protein (TN x 6.25), as requested by FAO for legal purposes (17). However, insect protein content is overestimated by this calculation because of chitin, a nitrogen-rich polysaccharide (51), as well as other non-protein components (71). To overcome the discrepancy between the calculated value considering TN x 6.25 value and the true protein content, insect-specific NPCF between 4.76 (72) and 5.41 (71) for whole TM, and between 5.00 (73) and 5.25 (71) for whole AD, were recently proposed. Overestimation of protein content has implications for protein quality assessment, as the IAA content per gram of protein are underestimated, which leads to lower DIAAS values and a consequent underestimation and potential misclassification of protein quality, which may particularly affect insects, as NPCF strongly differ from the statutory value of 6.25. For such products, the DIAAS calculation based on the summation of anhydrous AA as proposed earlier (27), would be more appropriate, because the uncertainty caused by non-protein nitrogen is avoided (74). Therefore, *in vitro* DIAAS values were established comparing both calculation methods, first based on crude protein (TN x 6.25, CP) and second by summing up anhydrous AA (sumAA) (Supplementary Table S3). As expected, the two methods yielded considerable differences in DIAAS dependent on the protein source.

### 4.3. Protein hydrolysis and digestibility

The protein hydrolysis of substrates during IVD was studied at the level of intact proteins, peptides, and release of short peptides and free amino acids. At the end of the IVD, no fully intact proteins were identified for any of the substrates, suggesting that all proteins are at least partly degraded during digestion. Subsequent investigation into the hydrolysis of specific proteins showed that each protein has an individual hydrolysis pattern, thus suggesting that changes in protein composition caused by processing may impact protein digestibility. As AD, TM, and chicken have comparable protein compositions, with many similar muscle proteins, total protein *in vitro* digestibility in the same range is not fully surprising. Moreover, digestion of *Actin* in AD and chicken occurred in a similar manner, suggesting that inter-species differences in protein sequence do not strongly affect its hydrolysis, at least for this specific protein. Chicken is generally accepted to be an easily digestible protein source, which was confirmed by *in vitro* IAA digestibility of 95.6 ± 0.7% in our model. Kashyap et al. (75) reported *in vivo* IAA digestibility of 92.0 ± 2.8% for chicken meat within a mixed-meal matrix measured in humans, using a dual stable isotope tracer method. Malla et al. (27) reported standardized ileal AA digestibility of diets formulated to contain 10% insect crude protein in pigs, which were broadly in the same range, even if generally lower than our *in vitro* digestibility of individual AA for both TM and AD. As this study employed commercially available insect powders, the potential processing and food preparation steps may have affected the standardized ileal AA digestibility. Further, as we assessed the *in vitro* digestibility of individual AA of insects in isolation, the presence of other ingredients in the mixed feed may have interacted with the proteins and digestive enzymes. Using rat bioassays with diets containing 10% crude protein, Poelaert et al. (76) found faecal true protein digestibility of 91.9 and 83.9% for TM and AD, respectively, and Jensen et al. (77) found true crude protein digestibility of 92% for freeze-dried TM. Caparros Megido et al. (78) used a different *in vitro* digestion model to assess different household cooking techniques, such as vacuum-cooking, frying, boiling, and oven-cooking for 15 or 30 min, for TM and found *in vitro* crude protein digestibility between 85.0 and 91.5%. Nonetheless, a comparison of these results from various methodologies and assays employing different species and food matrices is challenging, especially since the processing steps seem to have a strong influence on digestibility.

### 4.4. Processing and food preparation

Minimally treated TM and AD seem to be the best choice to preserve protein quality, as they were highly digestible and reached the highest *in vitro* DIAAS values in both TM and AD. Heat treatment is used to improve sensory quality and is often associated with improved protein digestibility due to the unfolded polypeptide chains, which are more accessible to the digestive enzymes (79). However, we did not observe this phenomenon with the oven-dried AD. Exposure to high temperatures (90°C) during the drying process probably caused the considerable reduction in total protein *in vitro* digestibility, which was possibly associated with the racemization of amino acid residues and inter- and intramolecular disulfide and Maillard reaction crosslinks (80, 81). Kinyuru et al. (82) also found a significantly reduced *in vitro* protein digestibility for grasshoppers (*Ruspolia differens*) that were toasted at 150°C for 5 min and solar dried at 30°C, in comparison to fresh grasshoppers. Bailey et al. (34) observed in a trial with gilts a decreased protein quality of ground beef after cooking at internal temperatures below 100°C, which was due to the lower standardized ileal digestibility of histidine and lysine, and they concluded that overcooking may reduce both AA digestibility and DIAAS.

The main purpose of reducing chitin from edible insects was to study the hypothesized negative effect of chitin on protein digestibility (33). While the chitin reduction itself was successful, the chitin-reduced substrates were no longer comparable to the initial substrates, as the composition of TM and AD shifted towards a higher fat and lower protein content, with substantial changes in protein composition and lower relative and absolute IAA content. It is therefore not possible to discern any potential effect of chitin on protein digestibility based on our digestibility results. Nonetheless, considering the high protein digestibility obtained by substrates without chitin reduction, and the rather low amount of chitin in these insect species, the removal of chitin may not be worthwhile, especially if the process results in IAA loss.

### 4.5. Sustainability vs. protein quality

High-quality protein from livestock is often associated with a major burden on the environment (34). Insect protein production has lower greenhouse gas emissions and requires similar amounts of energy and less land in comparison to milk, chicken, pork or beef. In addition, due to the relative novelty of large-scale insect rearing, further efficiency gains to improve productivity are more likely than in traditional livestock production (7). The high but lower DIAAS values of TM and AD (= 90, 0.5–3 years) in comparison to milk and meat proteins (≥ 100, 0.5–3 years) have to be balanced with the large difference in the reduced burden on the environment. Greenhouse gas emissions to produce legumes are slightly lower than edible insects (2), but proteins from legumes are often associated with lower digestibility (66, 75) due to antinutritional factors (37) and the plant cell wall structure (83), resulting in lower DIAAS values (< 90, 0.5–3 years) than TM or AD.

### 4.6. Strengths and limitations/implications for the future

The strengths of this study are: 1) the use of a previously validated and standardized *in vitro* model (20) based on the harmonized INFOGEST protocol (18); 2) the concomitant characterization of a widely consumed reference protein (chicken) as a reference for the insect proteins; and 3) the systematic assessment of several processing and food preparation methods, which can be applied to edible insects. Our study also has some limitations: 1) the *in vitro* model was previously validated for legumes, grains, and isolated proteins, and thus further *in vitro* and *in vivo* data are required to fully evaluate the nutritional quality of insect protein in human subjects; 2) the INFOGEST *in vitro* digestion is based on the physiological conditions of the digestion of adults, as requested by FAO (17). *In vitro* DIAAS based on the IAA requirements for children would be the most accurate, with an age- adjusted simulated digestion. Future research projects should be aimed towards adapting the INFOGEST protocol to simulate the digestion of other age groups, health conditions, and organisms; 3) the chitin reduction was achieved by a wet mechanical process, which also substantially reduced protein content and changed the protein and AA composition. It is therefore not possible to refute a potential inhibitory effect of chitin based on our *in vitro* data. An advantage of this *in vitro* model is the ability to screen a range of substrates at low cost and in a short time. This approach could be suited to optimize food processing in general to ensure protein quality, reducing the need for numerous animal- or human trials.

## 5. Conclusion

In conclusion, minimally processed blanched insects would be suitable candidates to potentially replace animal proteins from livestock, as they showed similar protein and AA compositions, comparable total protein *in vitro* digestibility and, albeit slightly lower, similar *in vitro* DIAAS values compared to chicken breast.

In general, TM and AD can be considered good-quality protein sources for all ages above 6 months based on *in vitro* DIAAS, when a suitable processing method is used. We show that the processing and food preparation of edible insects can influence the IAA profiles and *in vitro* digestibility, which may reduce protein quality. Further characterization of edible insects and research regarding the optimal processing methods for individual insect species, as well as the evaluation of protein quality within a full meal or as a finished product, such as a protein bar, will help to further refine evidence-based dietary recommendations for the human consumption of insects in the future.

## Data availability statement

The mass spectrometry proteomics data have been deposited to the ProteomeXchange Consortium via the PRIDE partner repository with the dataset identifier PXD041521 (56, 57). Original Mascot dat files were uploaded to the PRIDE proteomics identifications database (PXD041373) (56, 57).

## Ethics statement

Insects used in this study were obtained from Insekterei GmbH (registered in Switzerland as UID CHE-141.641.289) specialized in producing edible insects. The Swiss legislation does not require review and approval by an ethics committee because the insects were produced for human consumption according to Swiss food law, and available for sole in Swiss supermarkets.

## Author contributions

RP, DM, LH, and LE had primary responsibility for the final content, designed the research, and wrote the paper. LH, RP, PK, NH, LA-E, DM, and LE conducted the research, analyzed the data, and performed the statistical analyses. All authors contributed to the article and approved the submitted version.

## Funding

This study was funded by Agroscope, by Swiss Food Research (SFR Project1906), and by the Swiss National Research Foundation Practice to Science Program (Nr. PT00P3 199073 to DM, LH, and NH). Open access funding by Agroscope.

## Conflict of interest

The authors declare that the research was conducted in the absence of any commercial or financial relationships that could be construed as a potential conflict of interest.

## Publisher’s note

All claims expressed in this article are solely those of the authors and do not necessarily represent those of their affiliated organizations, or those of the publisher, the editors and the reviewers. Any product that may be evaluated in this article, or claim that may be made by its manufacturer, is not guaranteed or endorsed by the publisher.

## Figures and Tables

**Table:** 

AA	amino acids
AAA	aromatic amino acids
AD	*Acheta domesticus* (crickets)
DIAAR	digestible indispensable amino acid ratio
DIAAS	digestible indispensable amino acid score, lowest value of DIAAR
DM	dry matter
HPLC	High-performance liquid chromatography
IAA	indispensable amino acid
IVD	*in vitro* digestion
LC–MS	Liquid chromatography-mass spectrometry
NPCF	nitrogen-to-protein conversion factor
proxy DIAAR	DIAAR based on total protein digestibility
SAA	sulfur-containing amino acids
SDS-PAGE	sodium dodecyl sulfate polyacrylamide gel electrophoresis
TAA	total amino acids
TM	*Tenebrio molitor larvae* (mealworms)
TN	total nitrogen
