# Supplementary material for: Mealworm larvae (Tenebrio molitor) and crickets (Acheta domesticus) show high total protein in vitro digestibility and can provide good-to-excellent protein quality as determined by in vitro DIAAS
Source: Front Nutr. 2023 Jul 3;10:1150581. doi: 10.3389/fnut.2023.1150581 (PMC10350632; doi:10.3389/fnut.2023.1150581)
Supplement: Supplementary file 1 [file Data_Sheet_1.docx]

# ****Supplemental material****

# ****Fig. 1: Protein identifications by peptide mass fingerprinting****

**
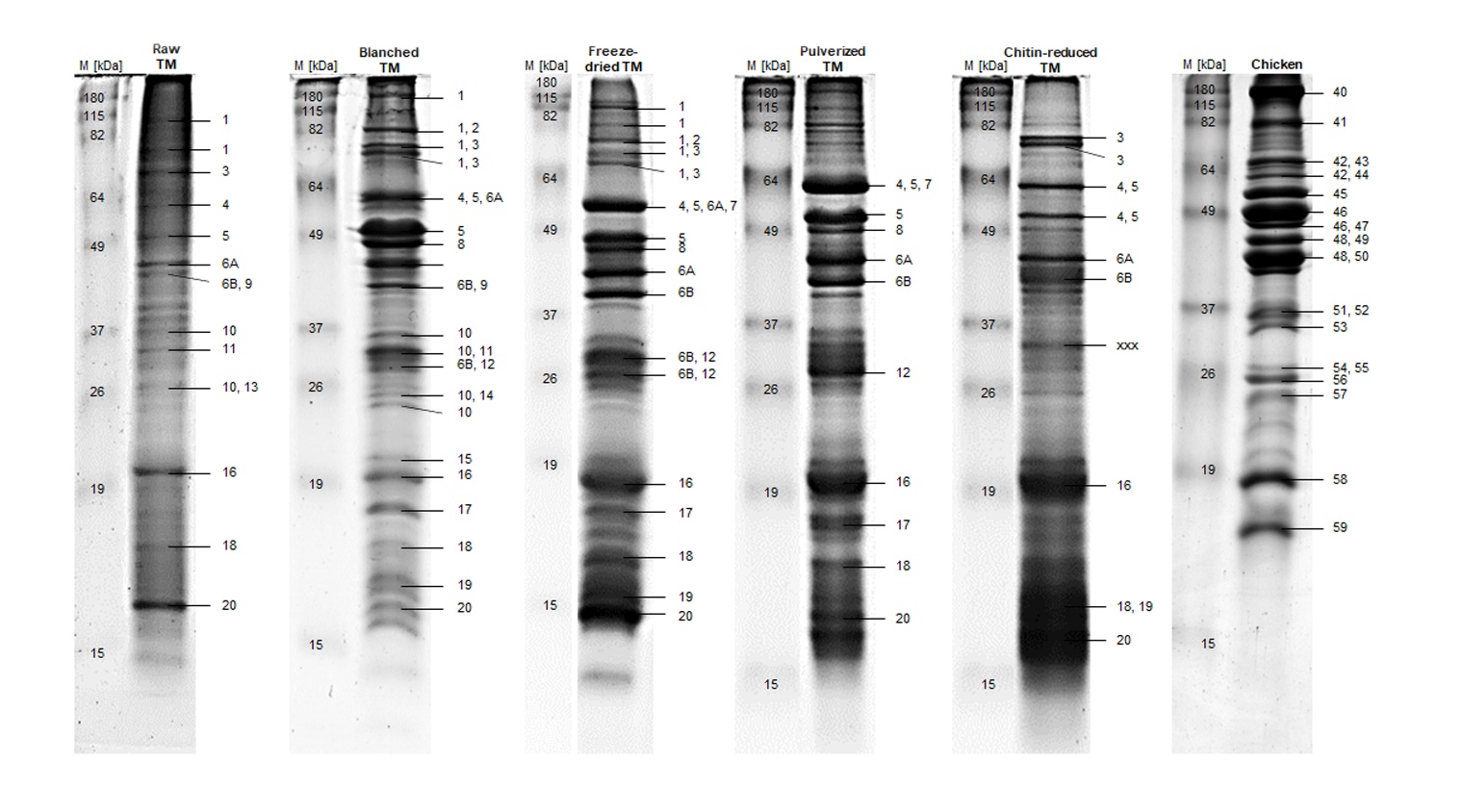
**

**Suppl. Fig.1a:** Gel bands are labeled by one or multiple numbers to indicate identified proteins, which are listed according to their numbers in Suppl. Table 1. TM = T.molitor larvae

**
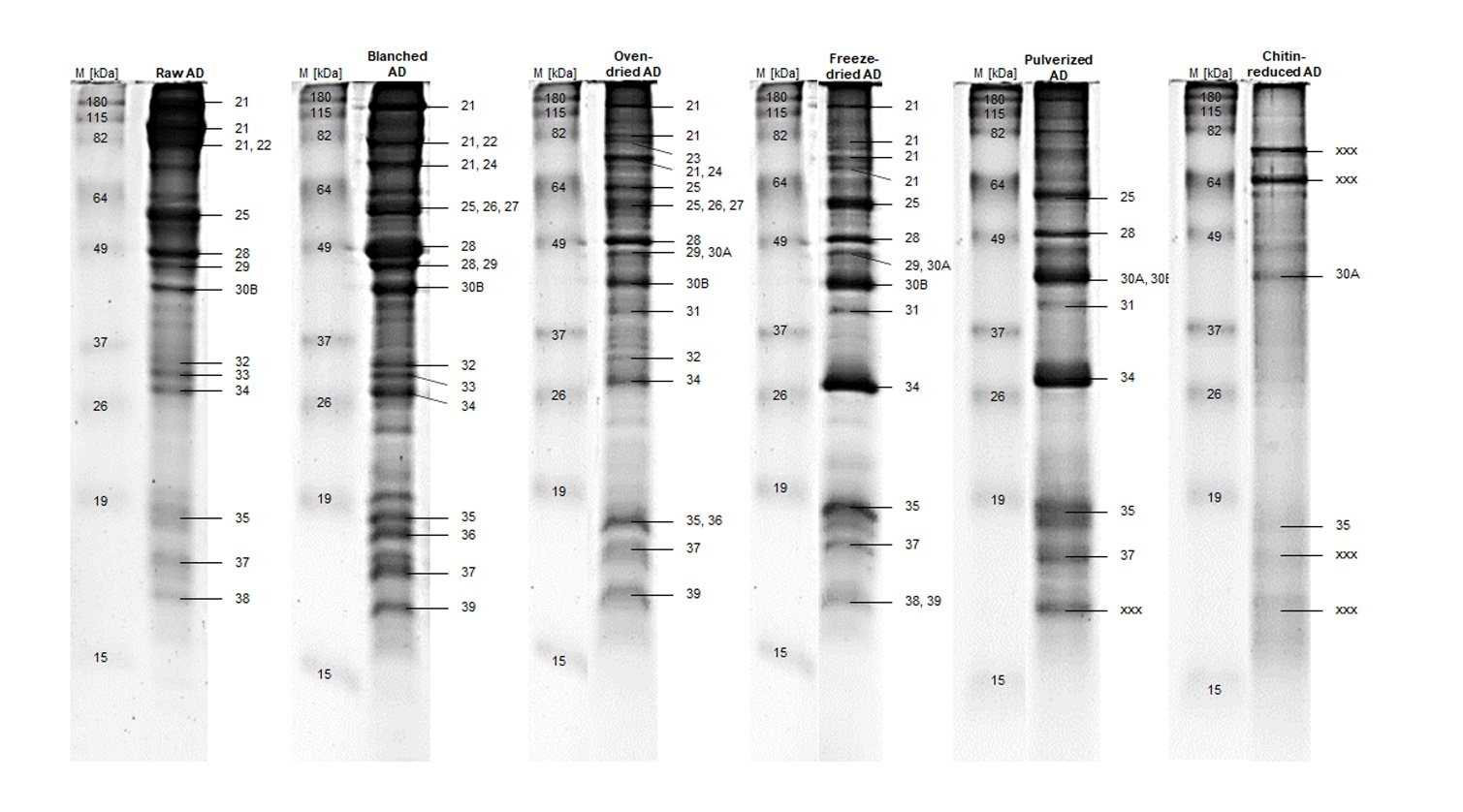
**

**Suppl. Fig.1b:** Gel bands are labeled by one or multiple numbers to indicate identified proteins, which are listed according to their numbers in Suppl. Table 1. AD = A.domesticus

**Suppl. Table 1**: Proteins identified by peptide mass fingerprinting after tryptic-in-gel digestion. The protein numbers correspond to gel band numbering in Suppl. Fig. 1A and 1B.

|  | Mass [Da] | Protein | Protein description |
| --- | --- | --- | --- |
|  | ***Tenebrio molitor larvae*** | | |
| 1 | 224465 | MYSA_DROME | Myosin heavy Chain, Muscle |
| 2 | 101976 | A0A0U2P8E2_9CUCU | Paramyosin |
| 3 | 90623 | Q9Y1W5_TENMO | 86 kDa early-staged Encapsulation-inducing Protein |
| 4 | 51240 | AMY_TENMO | Alpha-Amylase |
| 5 | 62445 | Q9Y1W6_TENMO | 56 kDa early-staged Encapsulation-inducing Protein |
| 6A | 32517 | A0A097P6H3_MONAT | Tropomyosin 1 |
| 6B | 32428 | A0A482D3R9_TENMO | Tropomyosin |
| 7 | 45713 | D6W953_TRICA | Troponin T, skeletal Muscle-like Protein |
| 8 | 41816 | ACT_MAYDE | Actin |
| 9 | 39550 | Q7YZB9_TENMO | Chitinase |
| 10 | 27968 | A1XG71_TENMO | Putative Serine Proteinase |
| 11 | 24803 | Q27013_TENMO | 28 kDa Desiccation Stress Protein |
| 12 | 21732 | D6WZU7_TRICA | Myosin regulatory light Chain 2-like Protein |
| 13 | 26074 | A1XG55_TENMO | Putative Trypsin-like Proteinase |
| 14 | 23551 | A0A077D0C3_TENMO | Glutathione S-Transferase sigma |
| 15 | 17278 | A0A140DKT3_9CUCU | Calponin (Fragment) |
| 16 | 65481 | Q7YZB8_TENMO | Cockroach Allergen-like Protein |
| 17 | 17027 | D6W9T6_TRICA | Myosin light Chain alkali-like Protein |
| 18 | 13171 | Q7YWD2_TENMO | 13 kDa hemolymph Protein a (Fragment) |
| 19 | 14738 | Q7YWC9_TENMO | 13 kDa Hemolymph Protein d |
| 20 | 14138 | Q7YWD7_TENMO | 12 kDa Hemolymph Protein b |
|  | ***Acheta domesticus*** | | |
| 21 | 224465 | MYSA_DROME | Myosin heavy Chain |
| 22 | 102750 | B3VTP0_BOMMO | Paramyosin |
| 23 | 83458 | E2IPC5_GRYFI | Heat Shock Protein 90 |
| 24 | 71431 | HSP7D_MANSE | Heat Shock 70 kDa Protein Cognate 4 |
| 25 | 46708 | A0A2P1ANK7_9ORTH | Troponin T |
| 26 | 50230 | TBB1_MANSE | Tubulin beta-1 Chain |
| 27 | 49908 | TBA1_DROME | Tubulin alpha-1 Chain |
| 28 | 41785 | E0VKP4_PEDHC | Actin, Muscle |
| 29 | 39859 | A0A455LAS4_9HYME | Arginine Kinase |
| 30A | 23039 | A0A4V1DVH3_ACHDO | Tropomyosin 2 (Fragment) |
| 30B | 23014 | A0A4P8D324_ACHDO | Tropomyosin 1 (Fragment) |
| 31 | 23079 | Q0Q613_GRYPE | Hypothetical accessory Gland Protein (Fragment) |
| 32 | 28096 | 1433Z_BOMMO | 14-3-3 Protein zeta |
| 33 | 23671 | A0A2P1ANL5_9ORTH | Troponin I |
| 34 | 22594 | Q49M29_GRYOR | Myosin light Chain |
| 35 | 19803 | Q16989_ACHDO | Apolipophorin-III |
| 36 | 15375 | H3C_BOVIN | Histone H3.3C |
| 37 | 13829 | B0WZ30_CULQU | Histone H2B |
| 38 | 16718 | B0WAK0_CULQU | Histone H2A |
| 39 | 11381 | H4_ACRAS | Histone H4 |
|  | ***Chicken breast*** | | |
| 40 | 223145 | MYSS_CHICK | Myosin heavy Chain, skeletal Muscle, adult |
| 41 | 104275 | ACTN2_CHICK | Alpha-Actinin-2 |
| 42 | 58014 | KPYK_CHICK | Pyruvate Kinase Muscle Isozyme |
| 43 | 66592 | Q2UZR2_CHICK | Phosphoglucomutase 1 |
| 44 | 47151 | CASQ1_CHICK | Calsequestrin-1 |
| 45 | 47196 | ENOB_CHICK | Beta-Enolase |
| 46 | 42051 | ACTS_CHICK | Actin, alpha skeletal Muscle |
| 47 | 43328 | KCRM_CHICK | Creatine Kinase M-type |
| 48 | 35703 | G3P_CHICK | Glyceraldehyde-3-phosphate Dehydrogenase |
| 49 | 4383 | Q92007_CHICK | Aldolase A (Fragment) |
| 50 | 32765 | TPM1_CHICK | Tropomyosin alpha-1 Chain |
| 51 | 31141 | TNNT3_CHICK | Troponin T, fast skeletal Muscle Isoforms |
| 52 | 28898 | PGAM1_CHICK | Phosphoglycerate Mutase 1 |
| 53 | 26620 | TPIS_CHICK | Triosephosphate Isomerase |
| 54 | 21683 | KAD1_CHICK | Adenylate Kinase Isoenzyme 1 |
| 55 | 242240 | Q9IAR9_CHICK | Structural Muscle Protein Titin (Fragment) |
| 56 | 20898 | MLE1_CHICK | Myosin light Chain 1, skeletal Muscle Isoform |
| 57 | 21234 | TNNI2_CHICK | Troponin I, fast skeletal Muscle |
| 58 | 18839 | MLRS_CHICK | Myosin regulatory light Chain 2, skeletal Muscle Isoform |
| 59 | 18375 | TNNC2_CHICK | Troponin C, skeletal Muscle |
|  |  |  |  |
| xxx | - | - | no identifications |

**Suppl. Table 2**: *In vitro* digestibility of individual AA, mean (AV) *in vitro* digestibility of AA and mean *in vitro* digestibility of IAA measured with TAA method. SD = standard deviation of N=3 experiments.

|  |  | TM | | | | AD | | | | | Chicken |
| --- | --- | --- | --- | --- | --- | --- | --- | --- | --- | --- | --- |
|  |  | Blanched | Freeze-dried | Pulverized | Chitin-reduced | Blanched | Oven-dried | Freeze-dried | Pulverized | Chitin-reduced | Chicken breast |
| HIS | AV | 100 | 98.9 | 97.0 | 93.6 | 96.0 | 77.7 | 90.8 | 91.7 | 85.1 | 100 |
|  | SD | 0.0 | 1.0 | 1.8 | 2.7 | 2.8 | 2.5 | 1.4 | 2.6 | 1.9 | 0.0 |
| SER | AV | 99.9 | 97.7 | 94.5 | 83.2 | 95.8 | 76.2 | 89.1 | 86.7 | 75.9 | 99.0 |
|  | SD | 0.2 | 2.6 | 2.8 | 7.5 | 2.9 | 4.7 | 1.5 | 2.5 | 4.5 | 0.9 |
| ARG | AV | 98.9 | 96.8 | 94.4 | 91.4 | 93.8 | 80.6 | 89.8 | 90.7 | 87.9 | 96.3 |
|  | SD | 0.9 | 2.5 | 2.2 | 3.0 | 1.7 | 4.2 | 1.2 | 1.8 | 1.3 | 1.1 |
| GLY | AV | 100 | 100 | 96.7 | 93.0 | 99.2 | 82.5 | 95.0 | 93.0 | 91.7 | 100 |
|  | SD | 0.0 | 0.0 | 3.1 | 6.6 | 1.0 | 2.7 | 2.1 | 3.5 | 2.2 | 0.0 |
| ASP | AV | 100 | 100 | 99.9 | 91.4 | 100 | 92.7 | 99.9 | 99.5 | 83.3 | 100 |
|  | SD | 0.0 | 0.0 | 0.3 | 5.4 | 0.0 | 4.3 | 0.1 | 0.8 | 4.0 | 0.0 |
| GLU | AV | 100 | 100 | 98.3 | 94.2 | 100 | 88.8 | 96.4 | 97.4 | 92.2 | 100 |
|  | SD | 0.0 | 0.0 | 1.7 | 2.6 | 0.0 | 3.6 | 1.0 | 1.3 | 1.4 | 0.0 |
| THR | AV | 99.7 | 97.4 | 94.3 | 82.7 | 96.2 | 79.3 | 89.7 | 91.5 | 78.2 | 98.9 |
|  | SD | 0.5 | 2.6 | 2.5 | 5.8 | 2.6 | 5.8 | 1.6 | 2.4 | 2.9 | 1.0 |
| ALA | AV | 97.9 | 95.6 | 95.0 | 91.5 | 84.2 | 72.7 | 86.7 | 80.3 | 90.0 | 98.3 |
|  | SD | 0.7 | 0.9 | 1.5 | 3.5 | 2.6 | 1.1 | 1.1 | 2.3 | 1.1 | 0.8 |
| PRO | AV | 97.7 | 95.6 | 95.5 | 97.0 | 87.9 | 74.7 | 87.8 | 83.5 | 90.2 | 99.5 |
|  | SD | 1.0 | 1.7 | 1.5 | 1.0 | 2.2 | 1.0 | 1.5 | 2.7 | 1.6 | 0.8 |
| LYS | AV | 100 | 99.8 | 97.0 | 91.0 | 99.5 | 85.7 | 94.4 | 96.1 | 85.2 | 99.3 |
|  | SD | 0.0 | 0.3 | 2.0 | 3.8 | 0.8 | 4.0 | 1.3 | 1.3 | 2.6 | 0.6 |
| TYR | AV | 91.6 | 89.3 | 85.8 | 84.5 | 79.7 | 61.4 | 81.0 | 69.0 | 65.7 | 89.3 |
|  | SD | 1.3 | 3.0 | 2.3 | 2.3 | 2.0 | 7.8 | 1.3 | 6.0 | 4.5 | 0.1 |
| CYS | AV | 99.9 | 93.6 | 87.7 | 59.6 | 99.1 | 74.5 | 85.7 | 88.0 | 62.4 | 92.1 |
|  | SD | 0.2 | 9.7 | 5.6 | 12.4 | 1.6 | 8.7 | 5.4 | 3.6 | 1.8 | 5.1 |
| MET | AV | 91.6 | 90.7 | 97.1 | 87.6 | 89.5 | 88.6 | 90.6 | 97.7 | 84.8 | 96.3 |
|  | SD | 0.3 | 1.3 | 2.3 | 4.6 | 1.2 | 2.8 | 2.3 | 0.1 | 9.1 | 2.3 |
| VAL | AV | 97.5 | 94.4 | 92.4 | 88.5 | 88.7 | 72.7 | 86.2 | 83.4 | 79.8 | 96.2 |
|  | SD | 1.1 | 2.2 | 2.3 | 3.8 | 2.5 | 3.3 | 1.2 | 2.1 | 1.6 | 1.6 |
| ILE | AV | 97.8 | 95.1 | 92.5 | 88.1 | 92.4 | 75.3 | 87.0 | 87.8 | 74.7 | 95.7 |
|  | SD | 0.9 | 2.5 | 2.7 | 4.0 | 2.8 | 3.5 | 1.2 | 1.9 | 2.2 | 1.4 |
| LEU | AV | 98.2 | 95.6 | 93.4 | 88.0 | 92.2 | 76.2 | 87.9 | 88.3 | 78.6 | 97.0 |
|  | SD | 0.9 | 2.1 | 2.1 | 4.0 | 2.3 | 3.1 | 1.1 | 2.1 | 2.3 | 1.1 |
| PHE | AV | 100 | 99.1 | 94.1 | 87.7 | 96.8 | 76.8 | 89.8 | 92.4 | 77.1 | 96.6 |
|  | SD | 0.0 | 1.1 | 2.8 | 4.5 | 2.8 | 4.6 | 1.3 | 2.3 | 2.9 | 1.5 |
| TRP | AV | 99.2 | 97.6 | 80.8 | 71.6 | 87.8 | 57.4 | 81.4 | 78.1 | 44.3 | 80.1 |
|  | SD | 1.4 | 4.1 | 19.9 | 26.1 | 10.6 | 27.9 | 6.1 | 20.5 | 23.3 | 0.5 |
| AA | AV | 98.6 | 96.8 | 95.2 | 90.7 | 93.3 | 79.0 | 90.3 | 89.5 | 85.4 | 98.3 |
|  | SD | 0.4 | 1.6 | 2.0 | 3.7 | 1.6 | 2.6 | 1.2 | 2.2 | 2.1 | 0.7 |
| IAA | AV | 98.2 | 96.5 | 93.2 | 86.5 | 93.2 | 76.6 | 88.7 | 89.7 | 76.4 | 95.6 |
|  | SD | 0.5 | 1.8 | 2.1 | 6.5 | 2.8 | 5.2 | 1.5 | 3.9 | 5.0 | 0.7 |

**Suppl. Table 3:** Overview of *in vitro* DIAAS and proxy *in vitro* DIAAS calculated for the three recommended amino acid scoring patterns by FAO 1) Infant (birth to 6 month); 2) Child (6 month to 3 year); and 3) Older child, adolescent, adult [1]; calculated with crude protein (TN x 6.25) or protein content based on AA (sum of anhydrous AA residues); and determined with either TAA analysis or R-NH_2_ analysis. Values are means (AV) ± SDs (n = 3) and first limiting amino acid is specified in parentheses. AD = *A.domesticus* (crickets), TM = *T.molitor larvae* (mealworms)

|  | | *In vitro* DIAAS | | Proxy *in vitro* DIAAS | | | |
| --- | --- | --- | --- | --- | --- | --- | --- |
|  |  | **(TAA^1^, sumAA^2^)** | **(TAA^1^, CP^3^)** | **(meanTAA^4^, sumAA^2^)** | **(meanTAA^4^, CP^3^)** | **(R-NH_2_^5^, sumAA^2^)** | **(R-NH_2_^5^, CP^3^)** |
| *Infant (birth to 6 month)* | |  |  |  |  |  |  |
| TM | blanched | 68.7 ± 1.0 (TRP) | 67.5 ± 0.9 (TRP) | 68.3 ± 0.3 (TRP) | 67.1 ± 0.3 (TRP) | 65.8 ± 1.9 (TRP) | 64.7 ± 1.8 (TRP) |
|  | freeze-dried | 74.0 ± 4.1 (SAA) | 64.1 ± 3.5 (SAA) | 74.1 ± 1.2 (TRP) | 64.3 ± 1.1 (TRP) | 71.1 ± 2.9 (TRP) | 61.6 ± 2.5 (TRP) |
|  | pulverized | 61.9 ± 15.2 (TRP) | 53.6 ± 13.2 (TRP) | 72.9 ± 1.5 (TRP) | 63.2 ± 1.3 (TRP) | 69.5 ± 1.3 (TRP) | 60.3 ± 1.1 (TRP) |
|  | chitin-reduced | 54.9 ± 4.7 (SAA) | 48.1 ± 5.5 (SAA) | 67.7 ± 2.8 (LEU) | 52.1 ± 2.1 (LEU) | 65.4 ± 0.4 (LEU) | 50.4 ± 0.3 (LEU) |
| AD | blanched | 52.1 ± 6.3 (TRP) | 45.9 ± 5.5 (TRP) | 55.4 ± 1.0 (TRP) | 48.8 ± 0.9 (TRP) | 53.2 ± 2.0 (TRP) | 46.8 ± 1.8 (TRP) |
|  | oven-dried | 36.0 ± 15.5 (TRP) | 32.1 ± 13.8 (TRP) | 49.5 ± 1.7 (TRP) | 44.1 ± 1.5 (TRP) | 42.4 ± 3.3 (TRP) | 37.8 ± 3.0 (TRP) |
|  | freeze-dried | 51.5 ± 3.8 (TRP) | 44.5 ± 3.3 (TRP) | 57.1 ± 0.8 (TRP) | 49.3 ± 0.7 (TRP) | 52.9 ± 1.7 (TRP) | 45.7 ± 1.5 (TRP) |
|  | pulverized | 49.4 ± 12.9 (TRP) | 42.7 ± 11.2 (TRP) | 56.6 ± 1.4 (TRP) | 49.9 ± 1.2 (TRP) | 52.0 ± 1.8 (TRP) | 45.0 ± 1.5 (TRP) |
|  | chitin-reduced | 27.4 ± 14.4 (TRP) | 20.2 ± 10.6 (TRP) | 55.1 ± 1.4 (LEU) | 38.3 ± 0.9 (LEU) | 53.9 ± 2.7 (LEU) | 37.4 ± 1.9 (LEU) |
|  | chicken breast | 64.9 ± 0.7 (TRP) | 58.6 ± 0.4 (TRP) | 72.6 ± 0.5 (AAA) | 65.6 ± 0.5 (AAA) | 73.9 ± 0.0 (AAA) | 66.8 ± 0.0 (AAA) |
|  |  |  |  |  |  |  |  |
| *Child (6 month to 3 year)* | |  |  |  |  |  |  |
| TM | blanched | 90.7 ± 0.1 (SAA) | 89.1 ± 0.1 (SAA) | 93.9 ± 0.4 (SAA) | 92.3 ± 0.4 (SAA) | 90.0 ± 2.6 (SAA) | 89.0 ± 2.5 (SAA) |
|  | freeze-dried | 90.4 ± 5.0 (SAA) | 78.4 ± 4.3 (SAA) | 95.1 ± 1.6 (SAA) | 82.4 ± 1.4 (SAA) | 91.2 ± 3.7 (SAA) | 79.1 ± 3.2 (SAA) |
|  | pulverized | 91.2 ± 2.3 (SAA) | 79.1 ± 2.0 (SAA) | 93.5 ± 1.9 (SAA) | 81.1 ± 1.7 (SAA) | 89.2 ± 1.6 (SAA) | 77.3 ± 1.4 (SAA) |
|  | chitin-reduced | 67.2 ± 7.6 (SAA) | 58.7 ± 6.7 (SAA) | 93.7 ± 3.8 (SAA) | 72.2 ± 3.0 (SAA) | 90.6 ± 0.6 (SAA) | 69.8 ± 0.4 (SAA) |
| AD | blanched | 104.2 ± 12.6 (TRP) | 91.8 ± 11.1 (TRP) | 107.0 ± 1.9 (SAA) | 94.3 ± 1.6 (SAA) | 102.6 ± 3.9 (SAA) | 90.4 ± 3.4 (SAA) |
|  | oven-dried | 72.0 ± 30.9 (TRP) | 64.2 ± 27.5 (TRP) | 85.0 ± 2.8 (LYS) | 75.7 ± 2.5 (LYS) | 72.8 ± 5.7 (LYS) | 64.8 ± 5.1 (LYS) |
|  | freeze-dried | 102.6 ± 3.4 (SAA) | 88.6 ± 2.9 (SAA) | 104.1 ± 1.4 (LYS) | 89.9 ± 1.2 (LYS) | 96.5 ± 3.2 (LYS) | 83.3 ± 2.7 (LYS) |
|  | pulverized | 98.8 ± 25.9 (TRP) | 85.4 ± 22.3 (TRP) | 103.1 ± 2.5 (LYS) | 89.1 ± 2.2 (LYS) | 94.9 ± 3.3 (LYS) | 81.9 ± 2.8 (LYS) |
|  | chitin-reduced | 54.8 ± 28.8 (TRP) | 40.4 ± 21.3 (TRP) | 80.2 ± 2.0 (LEU) | 55.7 ± 1.4 (LEU) | 78.3 ± 3.9 (LEU) | 54.4 ± 2.7 (LEU) |
|  | chicken breast | 125.4 ± 1.3 (AAA) | 113.3 ± 1.2 (AAA) | 127.8 ± 0.9 (LEU) | 115.5 ± 0.8 (LEU) | 130.1 ± 0.0 (LEU) | 117.5 ± 0.0 (LEU) |
|  |  |  |  |  |  |  |  |
| *Older child, adolescent, adult* | |  |  |  |  |  |  |
| TM | blanched | 106.4 ± 0.1 (SAA) | 104.7 ± 0.1 (SAA) | 110.2 ± 0.5 (SAA) | 108.4 ± 0.5 (SAA) | 106.3 ± 3.0 (SAA) | 104.5 ± 2.9 (SAA) |
|  | freeze-dried | 106.1 ± 5.9 (SAA) | 92.0 ± 5.1 (SAA) | 111.6 ± 1.9 (SAA) | 96.8 ± 1.6 (SAA) | 107.0 ± 4.4 (SAA) | 92.8 ± 3.8 (SAA) |
|  | pulverized | 107.1 ± 2.6 (SAA) | 92.8 ± 2.3 (SAA) | 109.8 ± 2.3 (SAA) | 95.2 ± 2.0 (SAA) | 104.7 ± 1.9 (SAA) | 90.8 ± 1.6 (SAA) |
|  | chitin-reduced | 78.8 ± 8.9 (SAA) | 69.0 ± 7.8 (SAA) | 106.5 ± 4.4 (LEU) | 82.0 ± 3.4 (LEU) | 102.9 ± 0.7 (LEU) | 79.3 ± 0.5 (LEU) |
| AD | blanched | 119.3 ± 3.0 (LEU) | 105.1 ± 2.6 (LEU) | 120.8 ± 2.1 (LEU) | 106.4 ± 1.9 (LEU) | 115.8 ± 4.4 (LEU) | 102.1 ± 3.9 (LEU) |
|  | oven-dried | 92.8 ± 39.8 (TRP) | 82.6 ± 35.5 (TRP) | 101.0 ± 3.4 (LYS) | 89.9 ± 3.0 (LYS) | 86.4 ± 6.8 (LYS) | 77.0 ± 6.1 (LYS) |
|  | freeze-dried | 115.0 ± 1.4 (LEU) | 99.4 ± 1.2 (LEU) | 118.2 ± 1.6 (LEU) | 102.1 ± 1.4 (LEU) | 109.6 ± 3.6 (LEU) | 94.6 ± 3.1 (LEU) |
|  | pulverized | 115.5 ± 2.7 (LEU) | 99.8 ± 2.3 (LEU) | 117.1 ± 2.9 (LEU) | 101.2 ± 2.5 (LEU) | 107.7 ± 3.7 (LEU) | 93.0 ± 3.2 (LEU) |
|  | chitin-reduced | 70.6 ± 37.2 (TRP) | 52.1 ± 27.4 (TRP) | 86.7 ± 2.1 (LEU) | 60.3 ± 1.5 (LEU) | 84.8 ± 4.2 (LEU) | 58.9 ± 3.0 (LEU) |
|  | chicken breast | 136.5 ± 1.5 (LEU) | 123.3 ± 1.3 (LEU) | 138.3 ± 1.0 (LEU) | 125.0 ± 0.9 (LEU) | 140.7 ± 0.0 (LEU) | 127.2 ± 0.0 (LEU) |

**^1^**TAA: *In vitro* digestibility of individual amino acids was assessed by TAA analysis to determine *in vitro* DIAAS.

**^2^**sumAA: Protein contents of protein sources were based on the summation of anhydrous amino acids to calculate *in vitro* DIAAS or proxy *in vitro* DIAAS.

**^3^**CP: Protein contents of protein sources were based on crude protein (total nitrogen x 6.25) to calculate *in vitro* DIAAS or proxy *in vitro* DIAAS.

**^4^**meanTAA: Mean of *in vitro* digestibility of individual AA (total protein *in vitro* digestibility), assessed by TAA analysis, was used to calculate proxy *in vitro* DIAAS.

**^5^**R-NH_2_: Total protein in vitro digestibility, assessed by R-NH_2_ analysis was used to calculate proxy *in vitro* DIAA

1. FAO, *Dietary protein quality evaluation in human nutrition. Report of an FAO Expert Consultation.*, in *FAO Food and Nutrition Journal*. 2013, Food and agriculture organization of the United Nations: Rome.
