# Supplementary material for: Mealworm larvae (Tenebrio molitor) and crickets (Acheta domesticus) show high total protein in vitro digestibility and can provide good-to-excellent protein quality as determined by in vitro DIAAS
Source: Front Nutr. 2023 Jul 3;10:1150581. doi: 10.3389/fnut.2023.1150581 (PMC10350632; doi:10.3389/fnut.2023.1150581)
Supplement: Supplementary file 2 [file Presentation_1.PPTX]

## Slide 1
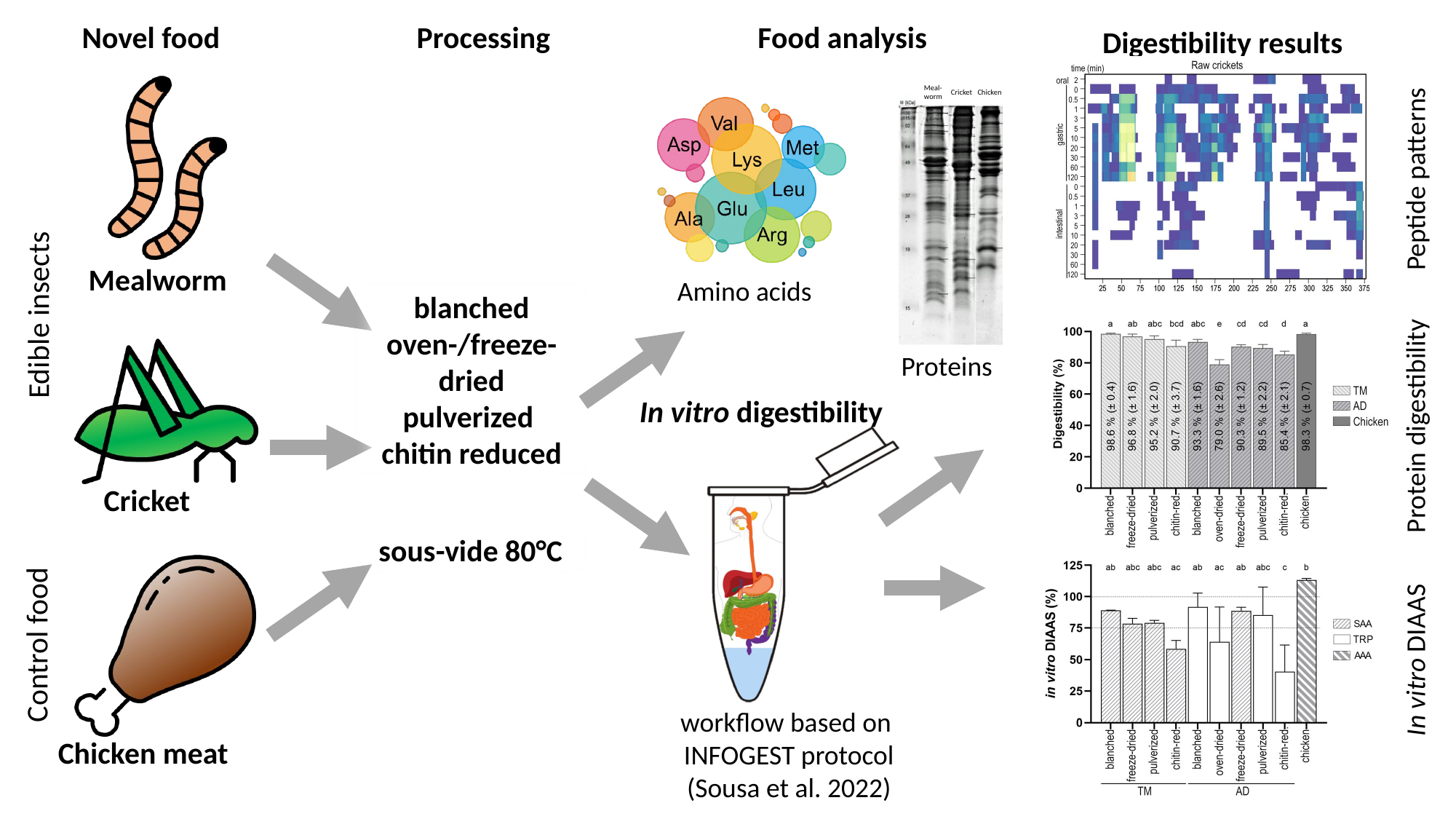

Novel food
Processing
Food analysis
Digestibility results
Meal-
worm
Cricket
Chicken
Peptide patterns
Mealworm
Amino acids
blanched
oven-/freeze-dried
pulverized
chitin reduced
Edible insects
Proteins
In vitro digestibility
 Protein digestibility
Cricket
sous-vide 80°C
Control food
In vitro DIAAS
workflow based on
INFOGEST protocol
(Sousa et al. 2022)
Chicken meat
